# Protocols for staining of bile canalicular and sinusoidal networks of human, mouse and pig livers, three-dimensional reconstruction and quantification of tissue microarchitecture by image processing and analysis

**DOI:** 10.1007/s00204-014-1243-5

**Published:** 2014-04-19

**Authors:** Seddik Hammad, Stefan Hoehme, Adrian Friebel, Iris von Recklinghausen, Amnah Othman, Brigitte Begher-Tibbe, Raymond Reif, Patricio Godoy, Tim Johann, Amruta Vartak, Klaus Golka, Petru O. Bucur, Eric Vibert, Rosemarie Marchan, Bruno Christ, Steven Dooley, Christoph Meyer, Iryna Ilkavets, Uta Dahmen, Olaf Dirsch, Jan Böttger, Rolf Gebhardt, Dirk Drasdo, Jan G. Hengstler

**Affiliations:** 1 Leibniz Research Centre for Working Environment and Human Factors (IfADo), TU Dortmund University, Dortmund, Germany; 2 Department of Forensic Medicine and Veterinary Toxicology, Faculty of Veterinary Medicine, South Valley University, Qena, Egypt; 3 Interdisciplinary Centre for Bioinformatics (IZBI), University of Leipzig, Leipzig, Germany; 4 Department of Digestive Surgery and Liver Transplantation, Trousseau Hospital, CHU, Tours, France; 5 Inserm U785 Unit, Centre Hépato-Biliaire, Paul Brousse Hospital, Villejuif, France; 6 Department of Visceral, Transplantation, Thoracic and Vascular Surgery, University Hospital Leipzig, Liebigstraße 21, Leipzig, Germany; 7 Molecular Hepatology - Alcohol Associated Diseases, Department of Medicine II, Medical Faculty Mannheim, Heidelberg University, Mannheim, Germany; 8 Scientific Databases and Visualization, HITS gGmbH, Schloss-Wolfsbrunnenweg 35, Heidelberg, Germany; 9 Experimental Transplantation Surgery, Department of General, Visceral and Vascular Surgery, Friedrich-Schiller-University Jena, Jena, Germany; 10 Institute of Pathology, Friedrich-Schiller-University Jena, Jena, Germany; 11 Institute of Biochemistry, Faculty of Medicine, University of Leipzig, Leipzig, Germany; 12 Unit Rocquencourt, INRIA, B.P.105, 78153 Le Chesnay, Cedex, France; 13 CNRS UMR 7598, Laboratoire Jacques-Louis Lions 4, France Université of Paris 06, pl. Jussieu, Paris, France

**Keywords:** Systems biology, Quantitative imaging, Confocal microscopy, Liver microarchitecture, Hepatocyte polarity

## Abstract

Histological alterations often constitute a fingerprint of toxicity and diseases. The extent to which these alterations are cause or consequence of compromised organ function, and the underlying mechanisms involved is a matter of intensive research. In particular, liver disease is often associated with altered tissue microarchitecture, which in turn may compromise perfusion and functionality. Research in this field requires the development and orchestration of new techniques into standardized processing pipelines that can be used to reproducibly quantify tissue architecture. Major bottlenecks include the lack of robust staining, and adequate reconstruction and quantification techniques. To bridge this gap, we established protocols employing specific antibody combinations for immunostaining, confocal imaging, three-dimensional reconstruction of approximately 100-μm-thick tissue blocks and quantification of key architectural features. We describe a standard procedure termed ‘liver architectural staining’ for the simultaneous visualization of bile canaliculi, sinusoidal endothelial cells, glutamine synthetase (GS) for the identification of central veins, and DAPI as a nuclear marker. Additionally, we present a second standard procedure entitled ‘S-phase staining’, where S-phase-positive and S-phase-negative nuclei (stained with BrdU and DAPI, respectively), sinusoidal endothelial cells and GS are stained. The techniques include three-dimensional reconstruction of the sinusoidal and bile canalicular networks from the same tissue block, and robust capture of position, size and shape of individual hepatocytes, as well as entire lobules from the same tissue specimen. In addition to the protocols, we have also established image analysis software that allows relational and hierarchical quantifications of different liver substructures (e.g. cells and vascular branches) and events (e.g. cell proliferation and death). Typical results acquired for routinely quantified parameters in adult mice (C57Bl6/N) include the hepatocyte volume (5,128.3 ± 837.8 μm^3^) and the fraction of the hepatocyte surface in contact with the neighbouring hepatocytes (67.4 ± 6.7 %), sinusoids (22.1 ± 4.8 %) and bile canaliculi (9.9 ± 3.8 %). Parameters of the sinusoidal network that we also routinely quantify include the radius of the sinusoids (4.8 ± 2.25 μm), the branching angle (32.5 ± 11.2°), the length of intersection branches (23.93 ± 5.9 μm), the number of intersection nodes per mm^3^ (120.3 × 103 ± 42.1 × 10^3^), the average length of sinusoidal vessel per mm^3^ (5.4 × 10^3^ ± 1.4 × 10^3^mm) and the percentage of vessel volume in relation to the whole liver volume (15.3 ± 3.9) (mean ± standard deviation). Moreover, the provided parameters of the bile canalicular network are: length of the first-order branches (7.5 ± 0.6 μm), length of the second-order branches (10.9 ± 1.8 μm), length of the dead-end branches (5.9 ± 0.7 μm), the number of intersection nodes per mm^3^ (819.1 × 10^3^ ± 180.7 × 10^3^), the number of dead-end branches per mm^3^ (409.9 × 10^3^ ± 95.6 × 10^3^), the length of the bile canalicular network per mm^3^ (9.4 × 10^3^ ± 0.7 × 10^3^ mm) and the percentage of the bile canalicular volume with respect to the total liver volume (3.4 ± 0.005). A particular strength of our technique is that quantitative parameters of hepatocytes and bile canalicular as well as sinusoidal networks can be extracted from the same tissue block. Reconstructions and quantifications performed as described in the current protocols can be used for quantitative mathematical modelling of the underlying mechanisms. Furthermore, protocols are presented for both human and pig livers. The technique is also applicable for both vibratome blocks and conventional paraffin slices.

**Electronic supplementary material:**

The online version of this article (doi:10.1007/s00204-014-1243-5) contains supplementary material, which is available to authorized users.

## Introduction

**Fig. 1:**
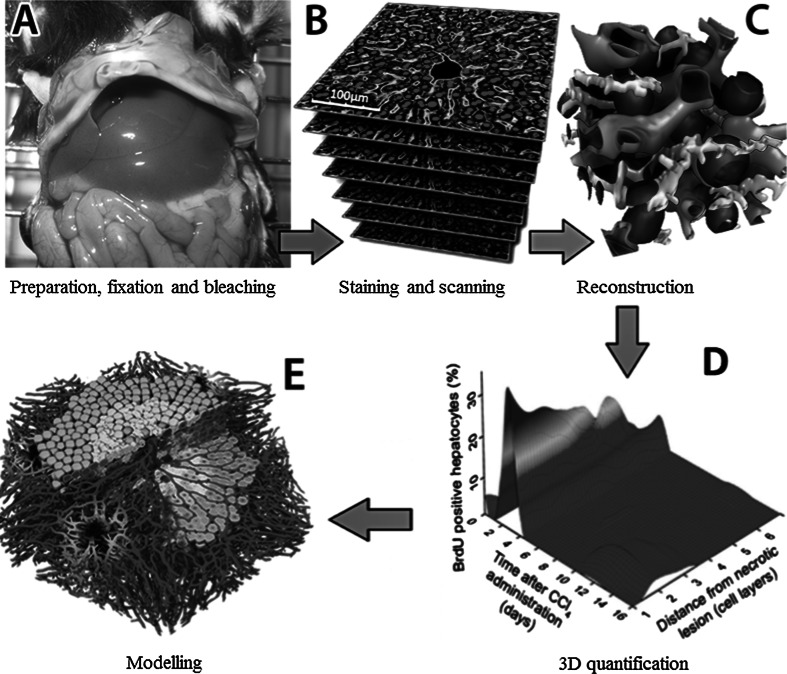
Imaging and analysis pipeline for reconstruction and quantification of liver microarchitecture

Current studies in both cell biology and hepatology largely rely on imaging and image analysis of 2D pictures. However, many parameters of cell and tissue architecture can be better quantified using 3D reconstructions (Hoehme et al. 2010; Braeuning et al. 2010). Unfortunately, the broad application of 3D imaging and image analysis is hampered by difficult-to-apply protocols (Godoy et al. 2013). To bridge this gap, we established methods for both imaging and automated analysis that encompasses staining, scanning, reconstruction, quantification and modelling (Fig. 1). Earlier versions of the described techniques and analysis pipeline have already been applied leading to the identification of key mechanisms of liver regeneration (Höhme et al. 2007; Hoehme et al. 2010; Schliess et al. 2014). This study primarily focuses on the reconstruction of sinusoidal networks. However, the current protocol also allows the simultaneous analysis of the intertwined sinusoidal and bile canalicular networks.

### Purpose of the protocols

#### Architectural staining

**Fig. 2:**
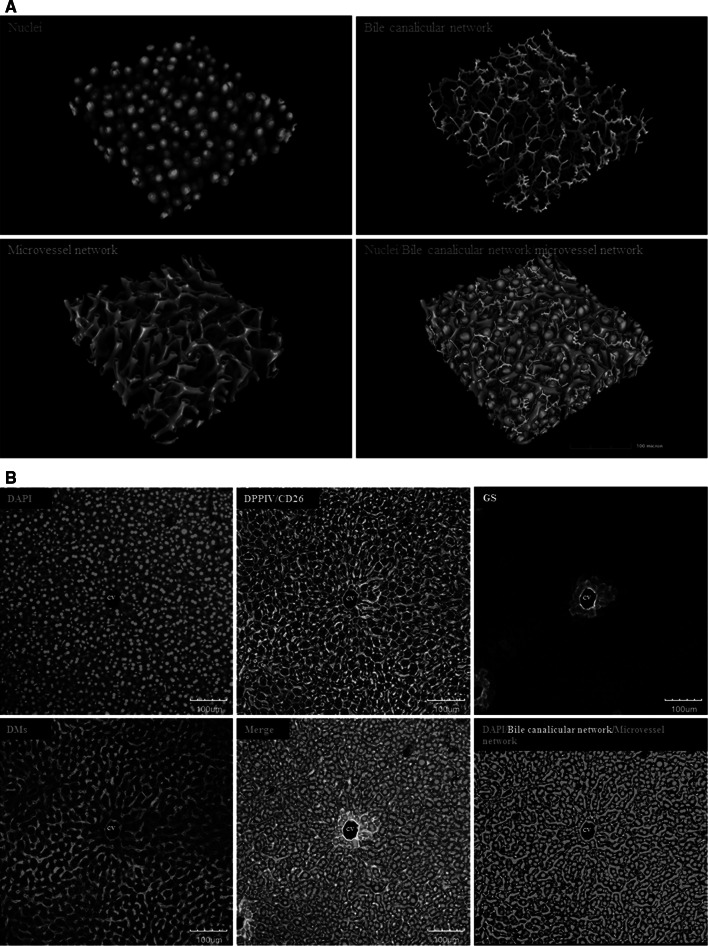
Liver architectural staining. **a** Examples of reconstructed mouse liver tissue. *Blue* nuclei, *green*: bile canalicular network and *red*: microvessel network. The *scale bars* are 100 μm. **b** Raw data obtained from a confocal laser scanning microscope. Only an individual slice level is shown. A routine *z*-stack of 75–100 μm includes approximately 180 such slice levels. *Blue* DAPI, *green* DPPIV/CD26, *red* DMs and *white* GS-positive hepatocytes. The *scale bars* are 100 μm. *CV* central vein, *DMs* donkey anti-mouse IgG, *DAPI* 4′,6-diamidino-2-phenylindole, *DPPIV* dipeptidyl peptidase IV, *GS* glutamine synthetase (colour figure online)

**Fig. 3:**
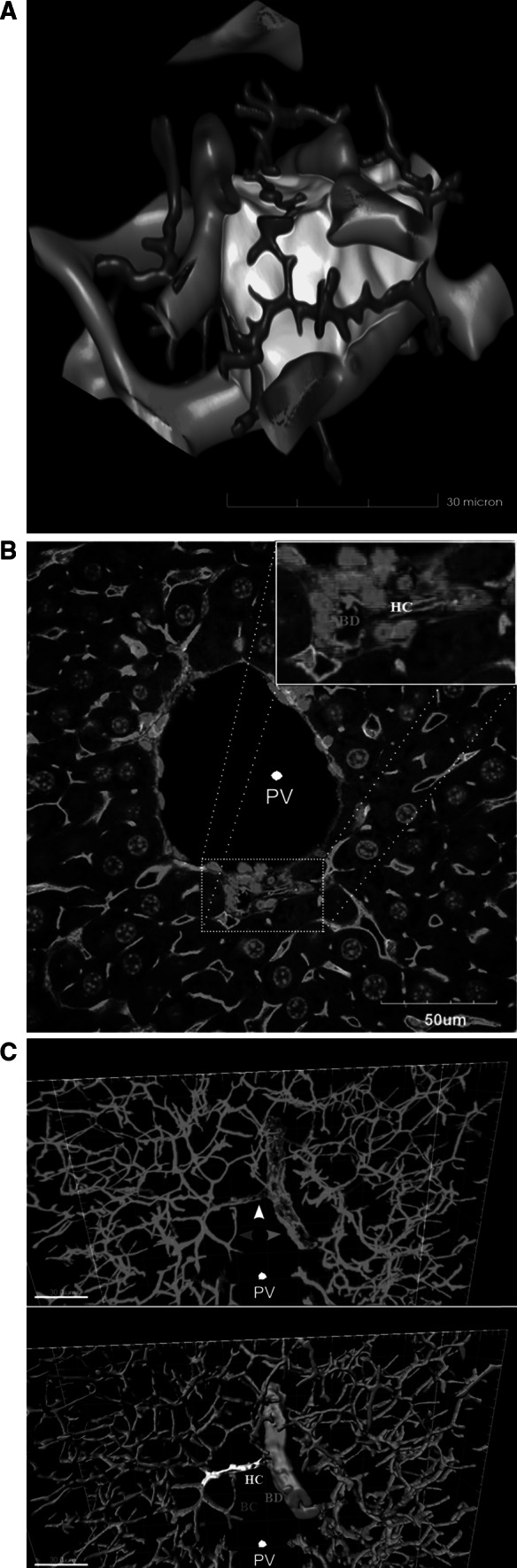
**a** Cell shape approximation of one hepatocyte (*yellow*) in relation to bile canaliculi (*green*) and sinusoids (*red*). Such reconstructions can be obtained for individual cells from the larger tissue blocks shown in Fig. 2a. The *scale bar* is 30 μm. **b** Individual slice level of a *z*-stack showing a bile duct and a canal of Hering in the upper right corner. The *scale bar* is 50 μm. **c** Reconstructions from the *z*-stacks level on the DPPIV/CD26 signal. The *upper panel* shows the reconstructed raw data, whereas the bile duct (*yellow*) and Hering canal (*white*) are highlighted in the *lower panel*. The *scale bars* are 30 μm. *BC* bile canaliculi, *BD* bile duct, *HC* Hering canal, *PV* portal vein (colour figure online)

 This protocol allows the visualization of bile canaliculi, sinusoidal endothelial cells and nuclei within the same tissue (Fig. 2). Moreover, glutamine synthetase (GS), an enzyme exclusively expressed in approximately two hepatocyte layers around the central vein (Fig. 2b), thus forming a ring around the central vein, is labelled to differentiate between central (GS positive) and periportal (GS negative) hepatocytes. The protocol describes the combined incubation of specific antibodies that directly target key architectural proteins of the liver. Anti-dipeptidyl peptidase IV/CD26 (DPPIV/CD26) is used as a marker of the bile canaliculi, which are visualized by green fluorescence. Donkey anti-mouse (DMs) IgG is used to localize the hepatic sinusoids, which is labelled with red fluorescence and appears yellow when merged with the green channel (Fig. 2b, merge). GS-positive hepatocytes around central veins are white (Fig. 2), and DAPI is used to counterstain the nucleus. From larger tissue blocks comprising entire liver lobules (Fig. 2), individual hepatocytes can be extracted, and their relation to the adjacent bile canaliculi and sinusoids visualized and quantified (Fig. 3a). Since DPPIV/CD26 is also expressed in bile duct epithelial cells, the technique is also adequate to visualize how bile ducts are linked to the bile canalicular network via the canals of Hering (Fig. 3b, c).

#### Staining for visualization of S-phases

**Fig. 4:**
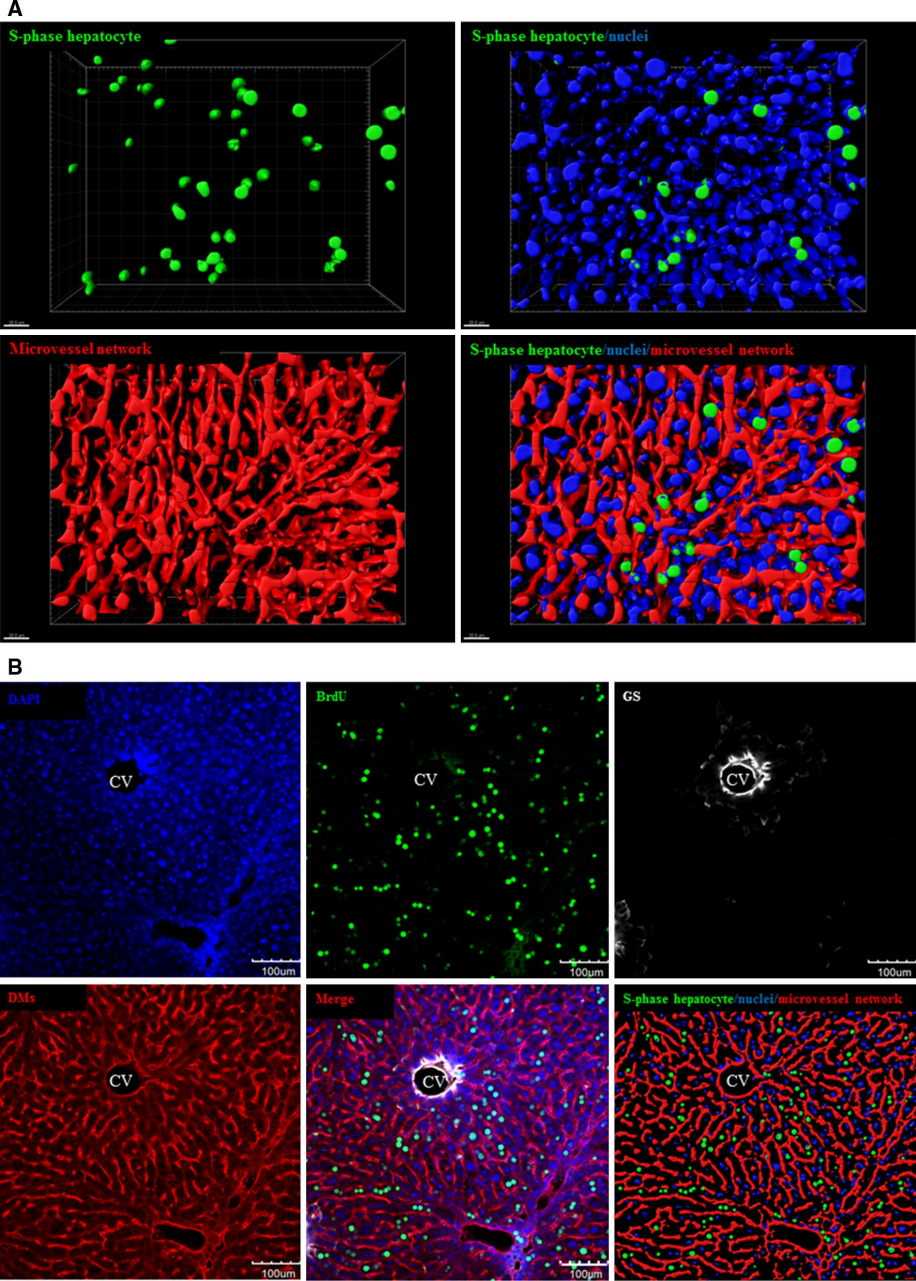
S-phase visualization staining. **a** Examples of reconstructed mouse liver tissue. The *green* nuclei are S-phase (BrdU) positive. *Blue*: nuclei, *green*: S-phase-positive nuclei and *red*: microvessel (sinusoidal) network. The *scale bars* are 20 μm. **b** Raw data were obtained from a confocal microscope. Only an individual slice level is shown. A routine *z*-stack of 75–100 μm includes approximately 180 such slice levels. *Blue* DAPI, *green* BrdU-positive nuclei, *red* DMs and *white* GS-positive hepatocytes. The *scale bars* are 100 μm. *BrdU* bromodeoxyuridine, *CV* central vein, *DAPI* 4′,6-diamidino-2-phenylindole, *DMs* donkey anti-mouse IgG, *GS* glutamine synthetase (colour figure online)

This technique allows for the differentiation between S-phase-positive and S-phase-negative nuclei (Fig. 4). It is based on the principle that cells incorporate BrdU into their DNA during S-phase, which can then be visualized using anti-BrdU antibodies. S-phase-positive nuclei appear green, whereas the S-phase-negative cells show only blue fluorescence due to DAPI staining. The sinusoidal endothelial cells appear red (Fig. 4), and the position of the central vein is identified by a ring of GS-positive hepatocytes (white).

### Image analysis protocols

Image analysis and quantification can be performed using our software TiQuant (www.msysbio.com/tiquant). This tool allows the quantitative analysis of key parameters of hepatocytes and the sinusoidal as well as bile canalicular networks (Table 1). The results in Table 1 are mean values and standard deviations from five mice (C57BL6/N, male, 8–12 weeks old). TiQuant quantifies large numbers of structures. For example, approximately 1.4 × 10^3^ hepatocytes, 450 first-order sinusoidal branches and 950 second-order bile canalicular branches are quantified in a single reconstructed tissue block at 20-fold magnification. These high numbers provide excellent conditions for statistical analyses. Moreover, all quantitative parameters of hepatocytes, sinusoids and bile canaliculi can be extracted from the same tissue specimen. Analysis of the ‘S-phase staining’ leads in a first step to the differentiation between BrdU-positive and BrdU-negative nuclei. As a result, further hierarchical quantifications in relation to different lobular structures are possible. For example, the fraction of BrdU-positive nuclei in relation to the distance of a necrotic lesion can be quantified (Fig. 1d). If this is performed in mice at different time intervals after intoxication with a hepatotoxic compound, spatial–temporal profiles can be obtained illustrating preferential lobular regions and periods of proliferation (Hoehme et al. 2010).

**Table 1:**
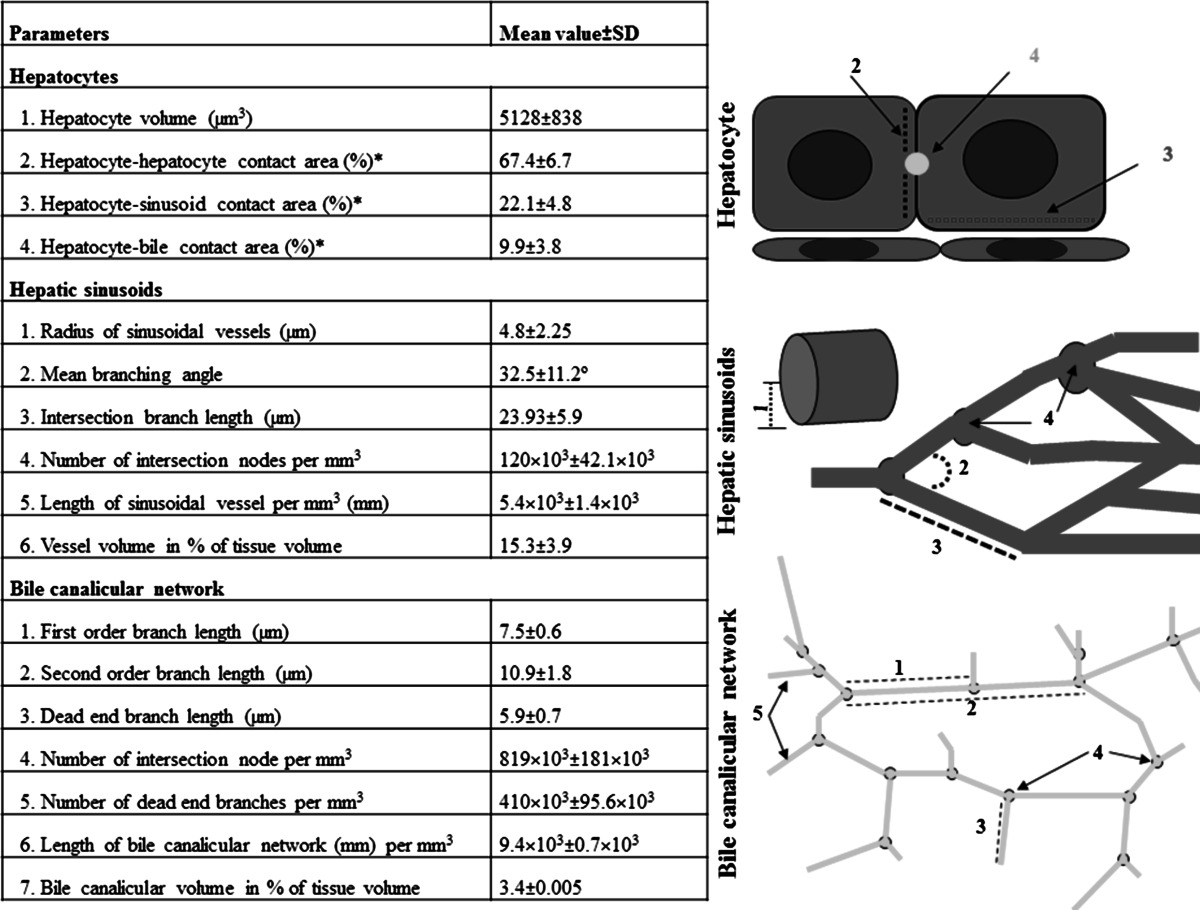
Parameters routinely quantified from the reconstruction shown in Fig. 2a

Numbers are mean ± standard deviation from livers of five male, adult C57 Bl6/N mice

* The three contact areas do not add up to 100 but only 99.4 % for experimental reasons, because the antibodies leave a small fraction (less than 1 %) of the hepatocyte surface unstained. Brief definition of some basic parameters: intersection node is a node in the network that is connected to more than two edges; first-order branch is a branch connecting two intersection nodes without disruption by a dead-end branch; second-order branch is a branch connecting two intersection nodes containing one or more than one dead-end branch in between; dead-end branch is a branch connected to the network by one end and unconnected at the other

### Advantages and limitations

The advantages of the described methods are: (1) bile canaliculi and sinusoids can be reconstructed from the same tissue block, which normally encompasses an entire lobule (*x*-and *y*-axis) and the margins of the neighbouring lobules (depending on the dataset); (2) besides entire lobules, detailed cell shape approximations and subcellular structures can also be visualized and quantified; (3) structures can be quantified in relation to their position within an individual lobule (e.g. periportal, mid-zonal and pericentral); (4) S-phase positive cells can be quantified in relation to their lobular position; (5) variants of the protocols with distinct antibody combinations have been optimized to visualize liver tissue from humans, mice and pigs and to reproducibly quantify their architectural parameters; (6) the staining protocols can be performed either manually or using an automated system for high throughput; and (7) the technique has been established for approximately 100-μm-thick vibratome slices. However, a modified version is also available for the staining of conventional paraffin sections.

A limitation of the technique is that staining vibratome slices thicker than 100 μm is still difficult with our technique due to limited antibody penetration. Therefore, the depth of the *z*-axis of the reconstructions is still smaller than the diameter of a liver lobule. Moreover, the resolution in the *z*-axis is worse when compared to the *x*- and *y*-axis. A further limitation of the described techniques is that they are more time consuming than conventional histology. Nevertheless, based on the confocal microscopy data, full 3D reconstructions and quantifications are obtained within a few hours per tissue block by applying the described protocols and software. Moreover, approximately 90 % of this time is fully automated and therefore can be parallelized which allows for high-throughput analyses if the computational power is available.

### Quality control and validation

**Fig. 5:**
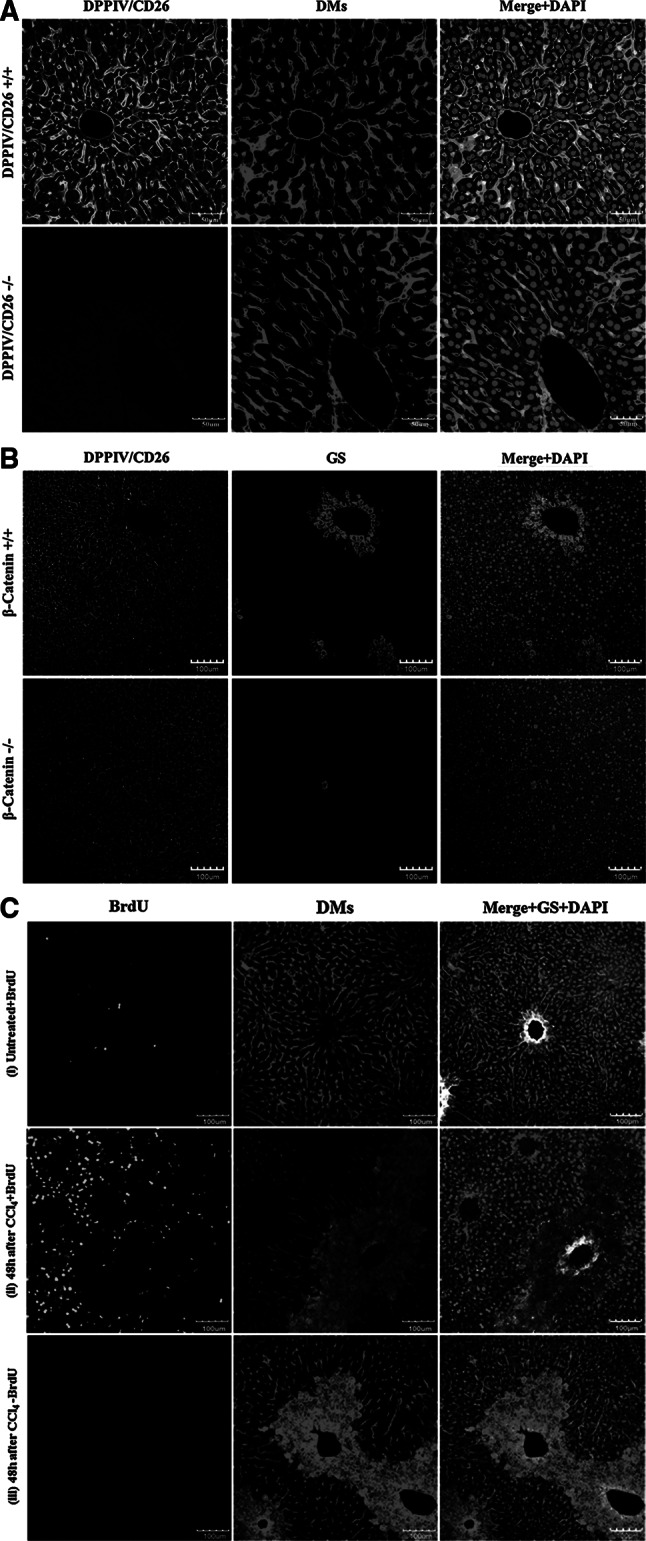
Validation and quality control of the antibodies. **a** The DPPIV/CD26 staining is completely abolished in DPPIV knockout mice (DPPIV/CD26−/−). The *scale bars* are 50 μm. **b** The signal of GS in a ring of pericentral hepatocytes is abolished in β-catenin knockout mice (β-catenin−/−). The *scale bars* are 100 μm. **c** The fraction of BrdU-positive nuclei (*green*) is low in control mice (1), high (approximately 30 %) in CCl_4_-exposed mice with BrdU injection (2) and absent in CCl_4_-treated mice without BrdU administration (3). The donkey anti-mouse IgG causes the *red* signal of the dead cell areas around the central veins. Therefore, this antibody can also be used to visualize necrotic regions. The *scale bars* are 100 μm. *BrdU* bromodeoxyuridine, *CCl*_*4*_ carbon tetrachloride, *DMs* donkey anti-mouse IgG, *DPPIV* dipeptidyl peptidase IV, *GS* glutamine synthetase (colour figure online)

**Fig. 6:**
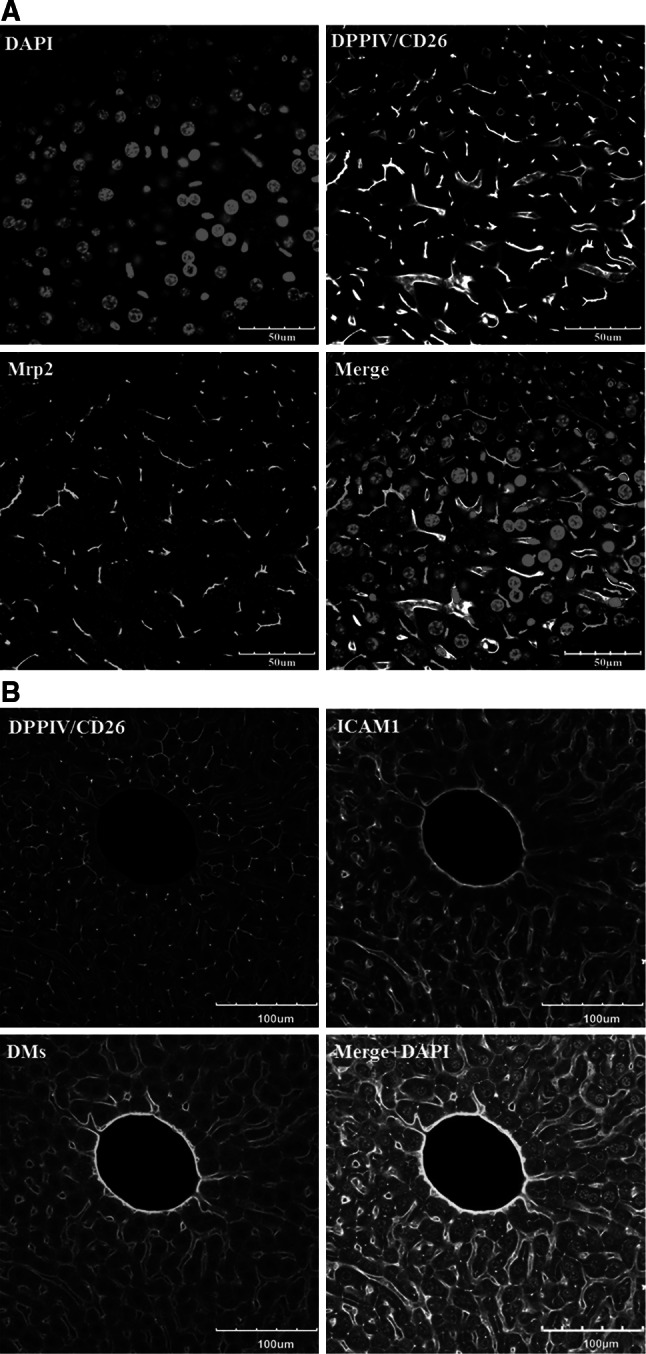
Validation of the DPPIV/CD26 and donkey anti-mouse IgG antibodies. **a** DPPIV/CD26 co-localizes with Mrp2. However, Mrp2 exclusively stains the bile canaliculi, whereas DPPIV/CD26 is also visible in the sinusoidal endothelial cells. The *scale bars* are 50 μm. **b** In architectural staining, donkey anti-mouse IgG (DMs) is used to visualize the sinusoidal endothelial cells. Co-staining with DMs (*red*) and the endothelial marker ICAM1 (*white*) shows good co-localization. The *scale bars* are 100 μm. *DMs* donkey anti-mouse IgG, *DPPIV* dipeptidyl peptidase IV, *ICAM1* intercellular adhesion molecule-1, *Mrp2* multi-drug resistance-associated protein 2 (colour figure online)

The technique for reconstruction of the bile canalicular and sinusoidal networks relies on an anti-mouse DPPIV/CD26 antibody from goat. Therefore, we investigated the specificity of this antibody using DPPIV/CD26 knockout mice (Fig. 5a). The negative result in the knockout mice demonstrates the specificity of the DPPIV/CD26 signal. The architectural staining protocol generates a yellow fluorescence signal for the sinusoidal endothelial cells (Fig. 5a, right panel, merge + DAPI). This signal reflects the merged green signal of DPPIV/CD26 and the red signal from the binding of the donkey anti-mouse IgG (DMs) antibody to the sinusoids. At first glance, this appears as an unnecessarily indirect way to visualize liver sinusoids, since antibodies are available that specifically stain the sinusoidal endothelial cells, such as anti-ICAM1 (Fig. 6b). However, the anti-ICAM1 antibody is obtained from rabbit and is therefore generated in the same species as the antibody against glutamine synthetase.

**Fig. 7:**
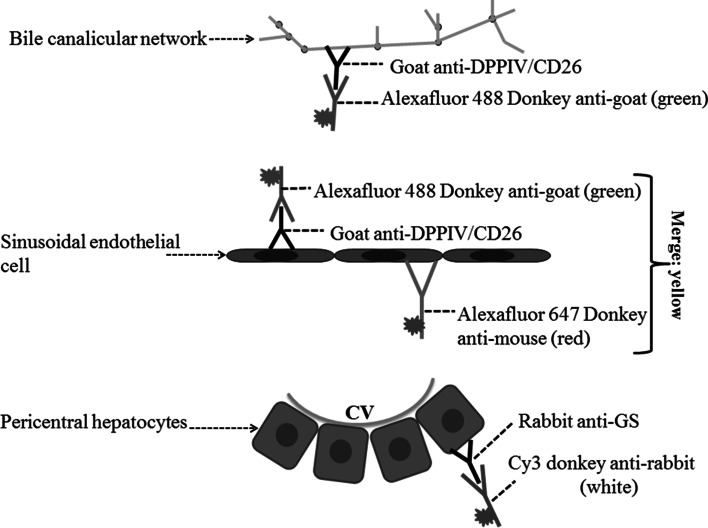
Principle of the ‘architectural staining’ for simultaneous visualization of the bile and sinusoidal networks as well as pericentral (GS positive) hepatocytes. *CV* central vein, *DPPIV* dipeptidyl peptidase IV, *GS* glutamine synthetase

In order to visualize all structures (bile canaliculi, sinusoids and GS-positive hepatocytes) in the same tissue, we present an optimized antibody combination—anti-DPPIV/CD26, anti-GS and secondary donkey anti-mouse IgG (DMs) antibodies. Anti-DPPIV/CD26 antibody labels both the bile canaliculi (Godoy et al. 2010; Hoehme et al. 2010) and to a lesser degree, the sinusoidal endothelial cells of mice, an observation that has previously been reported for both rats and mice (Rogler et al. 2007; Tarantola et al. 2012). To further validate the specificity of the DPPIV/CD26 antibody, we performed a co-staining with the bile canalicular multi-specific organic anion transporter 1 (Mrp2 or cMOAT). Both proteins were shown to co-localize at the bile canaliculi (Fig. 6a). However, in contrast to Mrp2, DPPIV/CD26 was additionally expressed in the sinusoidal endothelial cells of mice as mentioned above (Figs. 5a, 6a). Anti-DPPIV/CD26 antibody was nevertheless selected for our routine architectural staining, mainly due to its commercial availability. The anti-Mrp2 (against mouse) used in the present study was kindly provided by B. Stieger (supplemental table 1A), because the quality of all commercially available antibodies tested was inadequate for the current protocol. In our model, we used donkey anti-mouse IgG (DMs) to stain the endothelial sinusoids. We compared the staining signal of DMs to that of the endothelial-cell-specific antibody ICAM1 and observed a co-localization of the two signals (Fig. 6b). The principle of the architectural staining procedure can be based on the fluorescence of only two primary antibodies although three different colours (Fig. 7) visualize three different structures (bile canaliculi, sinusoids and pericentral hepatocytes). We initially tried to establish a technique based on three primary (anti-DPPIV/CD26, anti-ICAM1 or CD31 and anti-GS) and three secondary antibodies. However, this was not possible, because the 3 + 3 combination resulted in too high levels of background staining. In conclusion, the 2 + 3 staining strategy (principle is shown in Fig. 7) was the only technique based on commercially available antibodies that allowed the simultaneous and robust imaging of bile canaliculi, sinusoids and GS-positive hepatocytes.

To test the specificity of the anti-glutamine synthetase (GS) antibody, we used β-catenin knockout mice. Since GS expression is β-catenin dependent, these knockout mice are GS negative (Braeuning et al. 2010; Schreiber et al. 2011). No GS-positive ring of hepatocytes was observed around the central veins in the knockout mice (Fig. 5b), demonstrating the specificity of the anti-GS antibody. However, despite the absence of the GS-positive hepatocyte rings, a small number of solitary GS-positive hepatocytes were observed. This is in agreement with our previous publications (Braeuning et al. 2010; Schreiber et al. 2011) explaining both phenotype and growth behaviour of these residual β-catenin-positive hepatocytes.

To analyse the specificity of the anti-BrdU (bromodeoxyuridine) antibody, we treated mice as follows: (i) no carbon tetrachloride (CCl_4_); injection of 80 mg/kg BrdU (i.p.) 1 h before liver preparation, (ii) 1.6 g/kg CCl_4_ to induce liver damage (48 h before preparation) plus 80 mg/kg BrdU (i.p.) 1 h before liver preparation and (iii) 1.6 g/kg CCl_4_ (48 h before preparation) but no injection of BrdU (Fig. 5c). The result shows low levels of BrdU-positive nuclei in (i), high levels (approximately 30 % BrdU positive) in (ii) and a completely negative staining in (iii). This convincingly demonstrates specificity of the anti-BrdU antibody. Architectural staining of the CCl_4_-damaged liver tissue also results in a red fluorescent signal at the pericentral dead cell area (Fig. 5c, ii, iii), caused by the binding of the anti-DMs antibody to necrotic hepatocytes. This ‘wanted side effect’ represents another advantage of using the anti-DMs antibody.

### Application of the 3D protocol to further antibodies

Using the basic procedure described above for the ‘architectural staining’, the following already tested antibodies (suppl. Fig. 1) can be used with the same basic protocol by only exchanging the primary antibodies. The specific application conditions are outlined in supplemental Tables 1a, b. *Radixin* can be used as a marker of the bile canaliculi (suppl. Fig. 1A). As a constituent of myosin, radixin binds to the basic canalicular structure, whereas *DPPIV/CD26* and *Mrp2* represent functional bile canalicular proteins where loss of expression can occur, for example under conditions of inflammation. *β*-*Catenin* can be used as a marker of the basolateral membrane of all hepatocytes (suppl. Fig. 1A). *Collagen III* stains the fibrotic streets found in mice treated with a CCl_4_ fibrogenic protocol (Nussler et al. 2014). Labelling *collagen III* allows for the 3D reconstruction of fibrotic scars, which are rich in myofibroblasts (activated stellate cells). The latter can be visualized using *alpha*-*smooth muscle actin* (*α*-*SMA*) antibodies (Suppl. Fig. 1A). Two antibodies are particularly relevant for visualization of stellate cells; anti-*desmin* stains both activated and quiescent stellate cells while anti-*α*-*SMA* visualizes activated stellate cells only (suppl. Fig. 2). Both antibodies can be used to visualize the strong accumulation of stellate cells in damaged liver tissue (suppl. Fig. 2). *E*-*Cadherin* can be used as a marker for the basolateral membranes of periportal hepatocytes (suppl. Fig. 1B). The *low*-*density lipoprotein receptor (LDL*-*R)* also stains the basolateral membrane but the signal is homogenous throughout the liver lobule (suppl. Fig. 1B). Both *ICAM1* and lectin can be used to identify sinusoidal endothelial cells (suppl. Fig. 1B). While *ICAM1* is specific for sinusoidal endothelial cells, *lectin* additionally binds to the hepatocyte membrane and therefore represents a robust technique to visualize the surface of the hepatocyte. *Ki*-*67* and *alpha*-*tubulin* are both markers of cell cycle progression (suppl. Fig. 1C). While *Ki*-*67* is expressed by hepatocytes in G1-, S-, G2- and M-phases of the cell cycle, *alpha*-*tubulin* exclusively labels the mitotic spindle and can be used to analyse the spindle orientation in relation to the closest sinusoid or the apical membrane (Hoehme et al. 2010). Further markers are the tight junction protein *claudine 1* and the export pump *Mrp2*—both present in bile canaliculi (suppl. Fig. 1C).

### Application of the protocols to vibratome blocks of human and pig livers

**Fig. 8:**
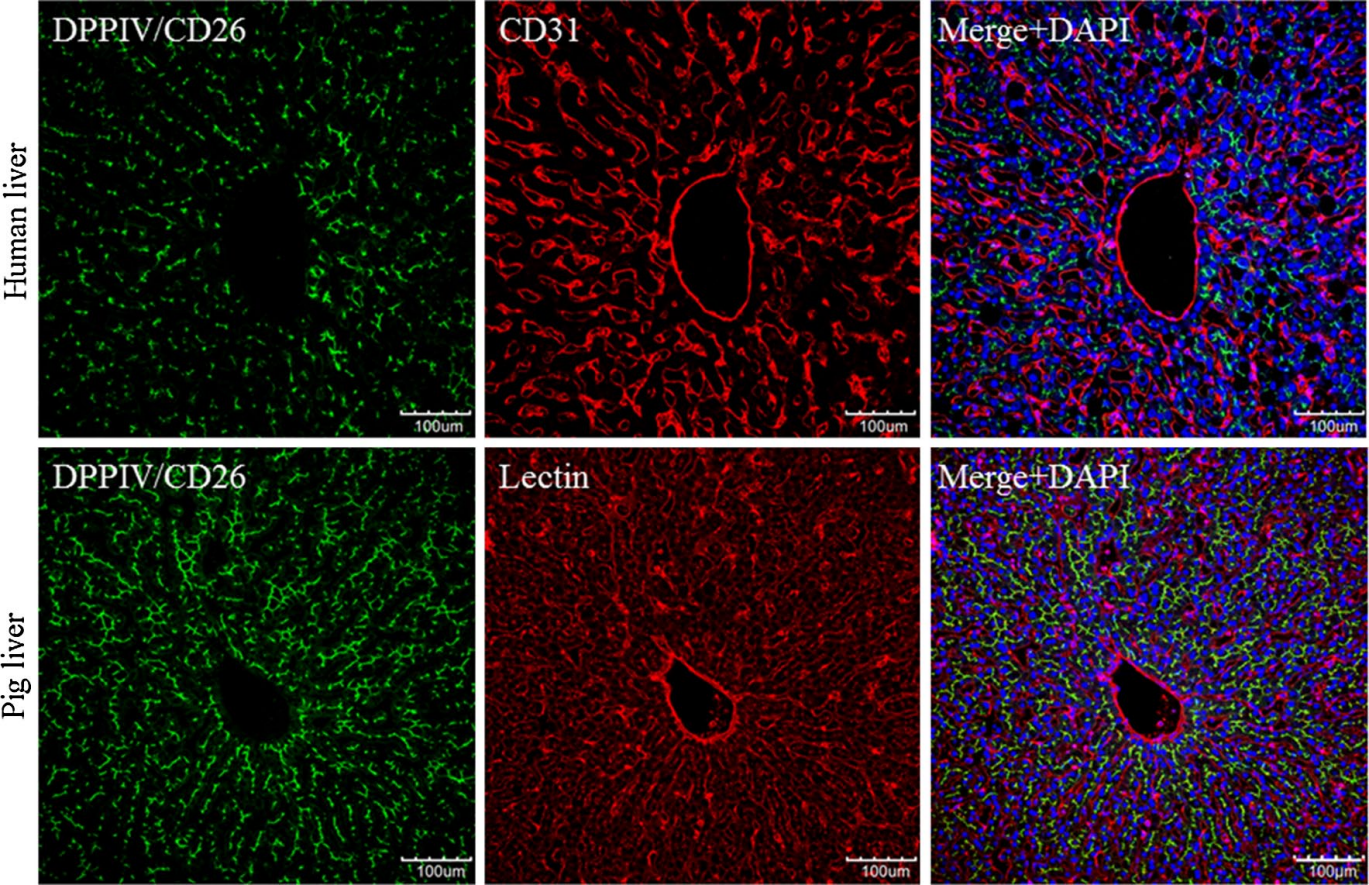
The staining and reconstruction techniques can also be applied for human and pig livers. The standard procedure ‘architectural staining’ can be used with the exception that the first and secondary antibodies have to be exchanged as described in supplemental Table 1a, b. It should be considered that the stainings shown in all other figures represent mouse livers. The *scale bars* are 100 μm

**Fig. 9:**
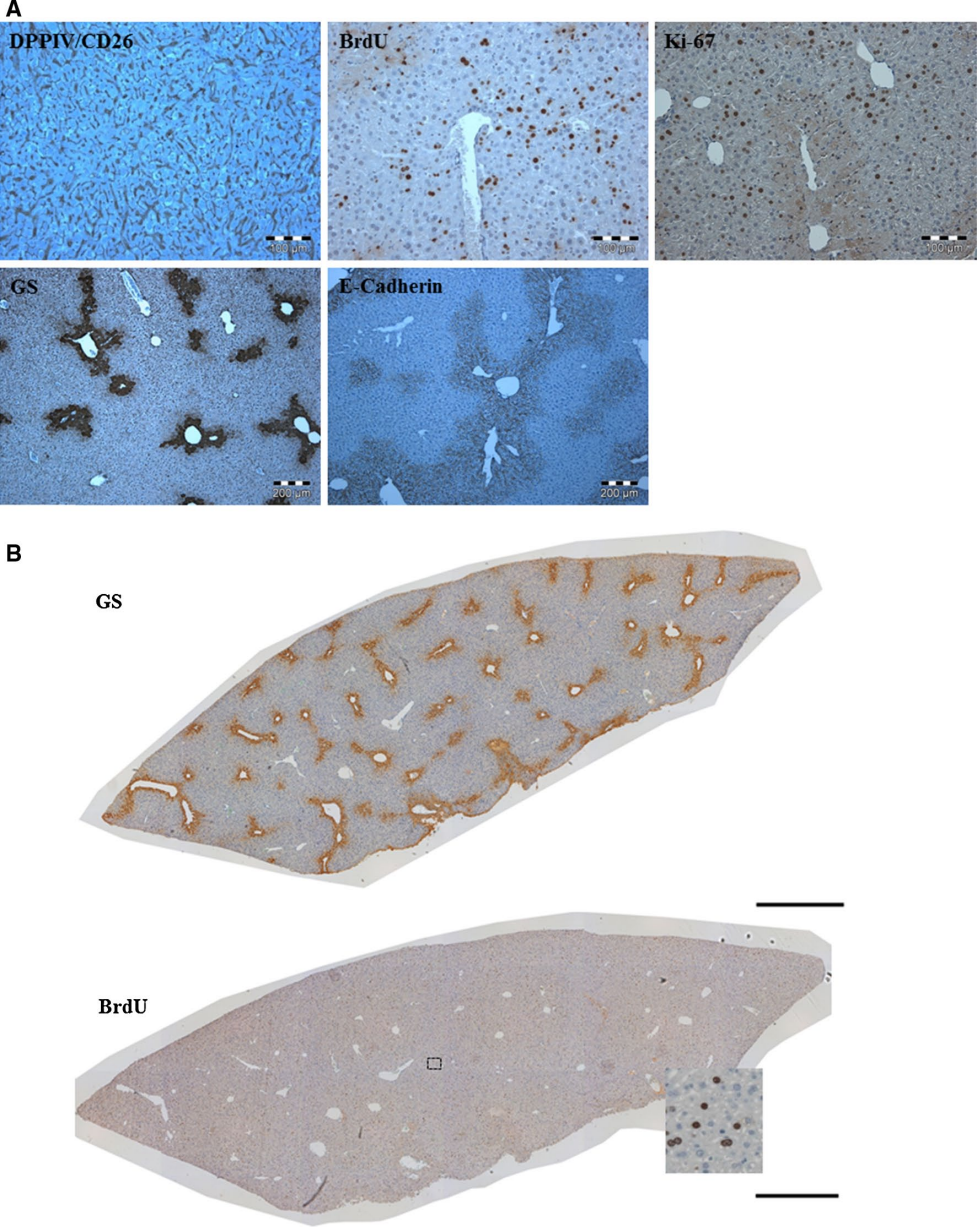
The protocols can also be applied to conventional paraffin slices with minor modifications as described under ‘protocol for immunostaining of paraffin sections’. **a** Immunostaining of DPPIV/CD26, BrdU, Ki-67, GS and E-Cadherin antibodies. *Scale bars* are 100 μm in the upper images and 200 μm in the lower. **b** Whole-slide scans of paraffin sections stained for GS or BrdU. *Scale bars* are 1,000 μm. *BrdU* bromodeoxyuridine, *DPPIV* dipeptidyl peptidase IV, *GS* glutamine synthetase

All analyses presented so far (Figs. 1, 2, 3, 4, 5, 6, 9) were performed using mouse livers. However, the ‘architectural staining’ protocol can also be applied with liver tissue from humans and pigs. The required antibodies and incubation conditions are summarized in supplemental tables 1A and 1B. The anti-human DPPIV/CD26 antibody from goat exclusively stains bile canaliculi in liver tissue of humans and pigs, but not sinusoidal endothelial cells (Fig. 8, upper left panel). The specificity is different in mice where DPPIV/CD26 is expressed in both the bile canaliculi and sinusoidal endothelial cells. To visualize the sinusoidal endothelial cells, an anti-human CD31 antibody from mouse is used (Fig. 8, upper middle panel). The merged image shows bile canaliculi in green and sinusoids in red (Fig. 8, upper right panel). Based on the described staining protocols, similar reconstructions and quantifications as described for mouse liver tissue can be applied to human and pig livers.

The anti-human DDPIV/CD26 antibody, used to detect the human protein, also recognizes pig DPPIV/CD26. Anti-human DPPIV/CD26 exclusively labels the bile canaliculi and not the sinusoidal endothelial cells (Fig. 8, lower left panel). Unfortunately, the anti-human CD31 is not suitable for pig liver (data not shown). Therefore, sinusoidal endothelial cells must be visualized using biotinylated lectin (Fig. 8, lower middle panel). The merged image shows both structures, bile canaliculi in green and the sinusoids in red (Fig. 8, lower right panel). Comparing architectural stainings from mice (Fig. 2), human and pigs (Fig. 8), it becomes obvious that the lumen of the human and pig sinusoids is larger compared with that of mice. A systematic quantification of interspecies differences of the liver microarchitecture will be addressed in a later publication.

### Application of the protocols to paraffin-embedded material

All the stainings presented so far have been done with approximately 100-μm-thick vibratome blocks (Figs. 1, 2, 3, 4, 5, 6, 8). The advantage of a vibratome block is the possibility to reconstruct 3D bile canalicular and sinusoidal networks. However, the standard ‘architectural staining protocol’ can be also applied to conventional paraffin slices. Minor modifications of the standard protocol are described under ‘protocol for immunostaining of paraffin sections’. A limitation of using paraffin sections is that only two-dimensional imaging is possible (Fig. 9a). Conversely, the sections can be advantageous because whole-slide tissue scans can be obtained as illustrated for GS and BrdU in Fig. 9b. In principle, such whole-slide scans offer the possibility of automated analysis of a very high number of structures leading to vastly improved statistical power.

## Protocols

All buffers and reagents are described under ‘general remarks and reagents’.

### Preparation of vibratome liver section for immunostaining

1. Fix liver lobes in Roti^®^-Histofix 4 % (CARL-ROTH, Karlsruhe, Germany, P087.1) for 3 days at 4 °C. Do not cut lobes into smaller pieces. Avoid squeezing of the liver tissue during preparation.
2. Store tissue sections in reagent B at 4 °C. In this reagent, liver tissue can be stored up to 6 months.
3. Immediately before staining, slice the liver lobe parallel to its surface using a vibrating blade microtome (Vibratome VT1000 S, Leica, Wetzlar, Germany) as follows:
  3.1. Fix the liver lobe in the specimen holder using glue (Histoacryl^®^Gewebekleber, B.Braun GmbH, Melsungen, Germany, 9381104) and wait for 2 min. Install the buffer tray and specimen holder in the vibratome.
  3.2. Pour reagent A into the buffer tray until the liver lobe is covered.
  3.3. Fix the vibratome feather blades (blades VT, Leica Microsystems, Wetzlar, Germany, 14020542056) tightly into the knife holder and install into the vibratome. Press the start button.
  3.4. Adjust the slicing settings to 75–100 μm thickness and low velocity.

### Protocol for liver architectural staining (example of a result: Figs. 2 and 3)

This protocol describes a staining technique for visualization of the liver microarchitecture including bile canalicular and sinusoidal networks. Moreover, the pericentral hepatocytes are identified by a positive GS signal, which allows localization of the central veins. Nuclei are stained with DAPI. A modified version for paraffin sections, using the same antibodies, is described in the subsequent section.

Samples: 75–100-μm vibratome liver sections that are stored in reagent B at 4 °C.

#### First day

1. Wash tissue sections three times for 10 min each in reagent A at room temperature.
2. Antigen de-masking:
  2.1 Add 400 ml of reagent C into a 500-ml plastic jar, cover and heat for 2 min in a microwave oven (Sharp Electronics, UK—95 °C).
  2.2 Pipette 2 ml of heated reagent C into each well of a 24-well tissue culture plate (SARSTEDT, Numbrecht, Germany, 83.1836).
  2.3 Remove tissue sections from reagent A and immerse in heated reagent C (in the 24-well tissue culture plate) for 2 min.
  2.4 During the 2-min incubation, reheat reagent C (left in the plastic jar) again for 2 min and repeat steps 2.2. and 2.3. for an additional 9 times.
  2.5 Cool tissue sections for 20 min at room temperature.
3. Wash tissue sections three times for 10 min each in reagent A at room temperature.
4. Blocking serum step:
  4.1 Pipette 1 ml of reagent E into each well of a 24-well tissue culture plate.
  4.2 Remove tissue sections from reagent A and immerse in reagent E.
  4.3 Incubate tissue sections in reagent E for 2 h at room temperature in a 24-well tissue culture plate.
5. Primary antibodies:
  5.1 Dilute rabbit anti-glutamine synthetase (Sigma-Aldrich, Missouri, USA, G2781, 1:2,000) and goat anti-mouse DPPIV/CD26 ectodomain (R&D systems, Minneapolis, MN, USA, AF954, 1:100) in reagent F in a 15-ml tube.
  5.2 Pipette 1 ml of the diluted antibodies (from step 5.1.) into each well of a 24-well tissue culture plate.
  5.3 Remove tissue sections from reagent E and immerse in the diluted antibodies.
  5.4 Incubate tissue sections in the diluted antibodies overnight at 4 °C.

#### Second day

6. Wash tissue sections three times for 10 min each in reagent A at room temperature.
7. Secondary antibodies:
  7.1 Dilute alexafluor^®^488-conjugated AffiniPure F(ab’)2 fragment donkey anti-goat IgG (H + L) (Dianova GmbH, Hamburg, Germany, 705-546-147, 1:100), Cy^TM^3-conjugated AffiniPure F(ab’)2 fragment donkey anti-rabbit (Dianova GmbH, Hamburg, Germany, 711-166-152, 1:200) and alexafluor^®^647-conjugated AffiniPure F(ab’)2 fragment donkey anti-mouse (DMs, Dianova GmbH, Hamburg, Germany, 715-606-150, 1:500) in reagent F in a 15-ml tube.
  7.2 Pipette 1 ml of the diluted antibodies (from step 7.1.) into each well of a 24-well tissue culture plate.
  7.3 Remove tissue sections from reagent A and immerse in the diluted antibodies.
  7.4 Incubate tissue sections in the diluted antibodies overnight at 4 °C.

#### Third day

8. Wash tissue sections three times for 10 min each in reagent A at room temperature.
9. Counterstaining:
  9.1 Prepare DAPI (4′,6-diamidin-2-phenylindol) solution as follows: To 10 ml of distilled water, add 1 μl of DAPI (Invitrogen, Darmstadt, Germany, D3571) and mix well.
  9.2 Pipette 1 ml of the diluted DAPI (from step 9.1.) into each well of a 24-well tissue culture plate.
  9.3 Remove tissue sections from reagent A and immerse in the diluted DAPI.
  9.4 Incubate tissue sections with DAPI for 90 min at room temperature.
10. Wash tissue section three times for 10 min each in reagent A at room temperature.
11. Wash tissue sections for 10 min in distilled water at room temperature.
12. Mount tissue sections on a microscope slide (Super frost plus, Thermo scientific, Gerhard Menzel GmbH, Braunschweig, Germany, Art. No. J1800AMNZ) using FluorPreserve reagent (Merck; Cat. No. 345787), cover with microscope cover glass (Thermo scientific, Gerhard Menzel GmbH, Braunschweig, Germany, 18 × 18 mm, 165027) and allow slides to dry in the dark.

### Protocol for S-phase visualization (example of a result: Fig. 4)

This protocol describes a staining technique for visualization and quantification of hepatocytes and non-parenchymal cells in S-phase. The protocol also visualizes the sinusoids and the pericentral hepatocytes (by GS staining). The nuclei are stained with DAPI.

Samples: 75–100-μm vibratome sections kept in reagent B at 4 °C.

#### First day

1. Wash tissue sections three times for 10 min each in reagent A at room temperature.
2. Antigen de-masking:
  2.1. Pipette 1.25 ml of reagent C into a 2-ml Eppendorf tube (Micro-tube, SARSTEDT, Numbrecht, Germany, 72.695.500) and immerse tissue sections in reagent C.
  2.2. Cook tissue sections in reagent C using a ThermoMixer (Model HTM 130L, HLC Biotech, Bovenden, Germany) at 95 °C for 25 min with shaking.
  2.3. Cool tissue sections for 20 min at room temperature.
3. Wash tissue sections three times for 10 min each in reagent A at room temperature.
4. HCl digestion:
  4.1. Pipette 2 ml of reagent D into each well of a 24-well tissue culture plate (SARSTEDT, Numbrecht, Germany, 83.1836).
  4.2. Remove tissue sections from reagent A and immerse in reagent D.
  4.3. Incubate tissue sections in reagent D for 10 min at room temperature.
5. Wash tissue sections three times for 10 min each in reagent A at room temperature.
6. Blocking serum step:
  6.1. Pipette 1 ml of reagent E into each well of a 24-well tissue culture plate.
  6.2. Remove tissue sections from reagent A and immerse in reagent E.
  6.3. Incubate tissue sections in reagent E for 2 h at room temperature in a 24-well tissue culture plate.
7. First Primary antibody:
  7.1. Dilute rat anti-BrdU, clone BU1/75 (ICR1) (AbD SEROTEC, Düsseldorf, Germany, MCA2060, 1:500) in reagent F in a 15-ml tube.
  7.2. Pipette 1 ml of the diluted antibodies (from step 7.1.) into each well of a 24-well tissue culture plate.
  7.3. Remove tissue sections from reagent E and immerse in the diluted antibodies.
  7.4. Incubate tissue sections in the diluted antibodies overnight at 4 °C.

#### Second day

8. Wash tissue sections three times for 10 min each in reagent A at room temperature.
9. First secondary antibody:
  9.1 Dilute alexafluor^®^488-conjugated AffiniPure F(ab’)2 fragment donkey anti-rat IgG (H + L) (Dianova GmbH, Hamburg, Germany, 712-546-150, 1:200) in reagent F in a 15-ml tube.
  9.2 Pipette 1 ml of the diluted antibodies (from step 9.1.) into each well of a 24-well tissue culture plate.
  9.3 Remove tissue sections from reagent E and immerse in the diluted antibodies.
  9.4 Incubate tissue sections in the diluted antibodies overnight at 4 °C.

#### Third day

10. Wash tissue sections three times for 10 min each in reagent A at room temperature.
11. Second primary antibody:
  11.1 Dilute rabbit anti-glutamine synthetase (Sigma-Aldrich, Missouri, USA, G2781, 1:2,000) in reagent F in a 15-ml tube.
  11.2 Pipette 1 ml of the diluted antibodies (from step 11.1.) into each well of a 24-well tissue culture plate.
  11.3 Remove tissue sections from reagent E and immerse in the diluted antibodies.
  11.4 Incubate tissue sections overnight at 4 °C.

#### Fourth day

12. Wash tissue sections three times for 10 min each in reagent A at room temperature.
13. Second secondary antibodies:
  13.1 Dilute Cy™3-conjugated AffiniPure F(ab’)2 fragment donkey anti-rabbit (Dianova GmbH, Hamburg, Germany, 711-166-152, 1:200) and alexafluor^®^647-conjugated AffiniPure F(ab’)2 fragment donkey anti-mouse (DMs, Dianova GmbH, Hamburg, Germany, 715-606-150, 1:500) in reagent F in a 15 ml tube.
  13.2 Pipette 1 ml of the diluted antibodies (from step 13.1.) into each well of a 24-well tissue culture plate.
  13.3 Remove tissue sections from reagent E and immerse in the diluted antibodies.
  13.4 Incubate tissue sections in the diluted antibodies overnight at 4 °C.

#### Fifth day

14. Wash tissue sections three times for 10 min each in reagent A at room temperature.
15. Counterstaining:
  15.1 Prepare DAPI (4′,6-diamidin-2-phenylindol) solution as follows: To 10 ml of distilled water, add 1 μl of DAPI (Invitrogen, Darmstadt, Germany, D3571) and mix well.
  15.2 Pipette 1 ml of the diluted DAPI (from step 15.1.) into each well of a 24-well tissue culture plate.
  15.3 Remove tissue sections from reagent A and immerse in the diluted DAPI.
  15.4 Incubate tissue sections with DAPI for 90 min at room temperature.
16. Wash tissue section three times for 10 min each in reagent A at room temperature.
17. Wash tissue sections for 10 min in distilled water at room temperature.
18. Mount tissue sections on microscope slides (Super frost plus, Thermo scientific, Gerhard Menzel GmbH, Braunschweig, Germany, Art. No. J1800AMNZ) using FluorPreserve reagent (Merck; Cat. No. 345787), cover with microscope cover glass (Thermo scientific, Gerhard Menzel GmbH, Braunschweig, Germany, 18 × 18 mm, 165027) and allow slides to dry in the dark.

### General remarks and reagents

#### General remarks

1. During slicing, discard the first and last tissue sections to avoid the margin of a tissue block where the structure often appears altered.
2. All incubation and washing steps should be done on a shaker (Type KL2, Edmund Buhler GmbH, Hechingen, Germany, Nr. 6115, approx. 200 movements).
3. Prepare all antibodies and DAPI directly prior to use.
4. Prepare antibodies, buffers and DAPI in 0.5-ml Eppendorf (Micro-tube, SARSTEDT, Numbrecht, Germany, 72.704.00) or 1.5-ml Eppendorf vials (Micro-tube, SARSTEDT, Numbrecht, Germany, 72.706) or in a 15-ml tube (SARSTEDT, Numbrecht, Germany, 62.554.502) or a 50-ml tube (SARSTEDT, Numbrecht, Germany, 62.547.254).
5. Incubate tissue sections overnight in 24-well tissue culture plates (SARSTEDT, Numbrecht, Germany, 83.1836.300).
6. Use 1 ml of reagent per well of a 24-well plate.

### Reagents

#### Reagent A (1× PBS)

1. For 5 l of 10× PBS:
  1.1 To 4 l of distilled water, add 10 g of potassium chloride (KCl, CARL-ROTH, Karlsruhe, Germany, Art.-Nr. 6781.1), 10 g of potassium dihydrogen phosphate (KH_2_PO_4_, CARL-ROTH, Karlsruhe, Germany, Art.-Nr. 3904.1), 400 g of sodium chloride (NaCl, CARL-ROTH, Karlsruhe, Germany, Art.-Nr.3957.2) and 46 g of disodium hydrogen phosphate anhydrous (Na_2_HPO_4_, CARL-ROTH, Karlsruhe, Germany, Art.-Nr. P030.2) and mix well to dissolve.
  1.2 Adjust the pH of the solution to 7.4.
  1.3 Bring the volume to 5 l with distilled water.
2. For 1 l of 1× PBS:
  2.1 To 900 ml of distilled water, add 100 ml of prepared 10× PBS and mix well.

#### Reagent B (preservation buffer)

1. To 1,000 ml of reagent A, add 300 g of glucose (D-(+)-glucose anhydrous, CARL-ROTH, Karlsruhe, Germany, Art.-Nr. X997.2) and mix well.
2. To 100 ml of the prepared glucose (in step 1), add 100 ml of Roti^®^-Histofix 4 % (CARL-ROTH, Karlsruhe, Germany, P087.1) and mix well.
3. Store reagent B at 4 °C.

#### Reagent C (antigen retrieval)

1. To 800 ml of distilled water, add 2.1 g of citric acid monohydrate (CARL-ROTH, Karlsruhe, Germany, Art. 3958.2) and mix well.
2. Adjust the pH of the solution to 6.0.
3. Bring the volume to 1 l with distilled water.

#### Reagent D (DNA denaturation agent)

1. Prepare 2N hydrochloric acid (HCl, 4 mol/l, CARL-ROTH, Karlsruhe, Germany, Art.Nr.NO76.1) as follows: To 10 ml of distilled water, add 10 ml of HCl and mix well.

#### Reagent E (blocking buffer)

1. To 97 ml of reagent A, add 3 g of bovine albumin fraction V, protease free (BSA, Serva Electrophoresis GmbH, Heidelberg, Germany, 11926, 100 g) and mix well.
2. Add 3 ml of Tween80 (Sigma-Aldrich, Schnelldorf, Germany, P8074, 500 ml) and mix well.
3. Prepare 10 ml aliquots and store at −20 °C.

#### Reagent F (dilution buffer)

1. To 97 ml of 1× PBS, add 0.3 g of bovine albumin fraction V, protease free (BSA, Serva Electrophoresis GmbH, Heidelberg, Germany, 11926, 100 g) and mix well.
2. Add 3 ml of Tween80 (Sigma-Aldrich, Schnelldorf, Germany, P8074, 500 ml) and mix well.
3. Prepare 10 ml aliquots and store at −20 °C.

### Protocol for immunostaining of paraffin sections (example of a result: Fig. 9)

This protocol describes a staining technique for paraffin liver sections.

Samples: 5–8 μm paraffin-embedded sections.

1. De-paraffinize the sections in four washes of roti-histol (Carl-ROTH; Karlsruhe-Germany; Art. No. 6640) for 5 min each.
2. Rehydrate the sections using descending ethanol (Carl-ROTH; Karlsruhe-Germany; Art. No. 9065.4) gradients (100, 95, 90, 70, 50 and 30 % ethanol, for 5 min each).
3. Wash the sections once in distilled water for 5 min.
4. Unmask the antigen of interest using an acid and heat treatment as follows:
  4.1 Place the sections in a container, cover with freshly prepared reagent C and heat at 95 °C for 7 min in a microwave oven (Sharp Electronics, UK).
  4.2 Top off with reagent C and reheat at 95 °C for 7 min.
  4.3 Allow the sections to cool in the heated reagent C for approximately 20 min.
5. Wash the sections in reagent A three times for 5 min each.
6. Block the endogenous peroxidase by a methanolic hydrogen peroxide (H_2_O_2_) treatment. For 100 ml of 30 % H_2_O_2_ (Carl-ROTH; Karlsruhe-Germany; Art. No. 8070.4), add 100 ml of methanol (J.T.Baker; Griesheim-Germany; JT9093-3) and incubate the sections in the methanolic H_2_O_2_ for 30 min at room temperature in a dark box.
7. Wash the sections in reagent A three times for 5 min each.
8. Block any unspecific binding using reagent E. Remove the sections from reagent A and immerse in reagent E. Incubate the sections in reagent E for 1 h at room temperature in a humified chamber.
9. Drain the blocking solution from the slides and apply two drops of avidin solution (Avidin biotin blocking kit; Vector Laboratories; Dossenheim-Germany; SP2001) for 15 min.
10. Remove the avidin solution from the slides and apply two drops of biotin solution (Avidin biotin blocking kit; Vector Laboratories; Dossenheim-Germany; SP2001) for 15 min.
11. Drain the biotin solution from slides.
12. Apply approximately 200 μl of the diluted primary antibody (in reagent F) at the recommended concentration per slide (supplemental table 1A) and incubate the sections overnight at 4 °C.
13. Wash the sections in reagent A three times for 5 min each.
14. Apply approximately 200 μl of the diluted secondary biotinylated antibody (in reagent F) at the recommended concentration per slide (supplemental table 1B) and incubate the sections for 60 min.
15. Wash the sections in reagent A three times for 5 min each.
16. Apply approximately 200 μl of the diluted horse-radish peroxidase (in reagent F) at the recommended concentration per slide (Supplemental Table 1B) and incubate the sections for 60 min.
17. Wash the sections in reagent A three times for 5 min each.
18. Prepare the 3,3′-diaminobenzidine (DAB) peroxidase substrate (Vector Laboratories; Dossenheim-Germany; SK-4100) as follows:
  18.1 To 5.0 ml of distilled water, add 2 drops of the buffer stock solution and mix well.
  18.2 Add 4 drops of the DAB stock solution and mix well.
  18.3 Add 2 drops of the hydrogen peroxide solution and mix well.
  18.4 Incubate the sections with the substrate working solution at room temperature for 4 min.
  18.5 Wash the sections once in distilled water for 5 min.
19. Counterstain the sections in Mayer’s haematoxylin (Merck, Langenfeld-Germany; art. Number: 1092492500) for 120 s and immediately wash the sections under tap water for 10 min.
20. Dehydrate the sections using ascending ethanol (Carl-ROTH; Karlsruhe-Germany; Art. No. 9065.4) gradients (30, 50, 70, 90, 95 and 100 % ethanol, for 10 s each).
21. Wash the sections twice in roti-histol (Carl-ROTH; Karlsruhe-Germany; Art. No. 6640) for 3 min each.
22. Mount the sections on microscope slides (Super frost plus, Thermo scientific, Gerhard Menzel GmbH, Braunschweig, Germany, Art.No.J1800AMNZ) using Entellan (Merck-Millipore, Darmstadt-Germany; Cat. No. 1079600500), cover with microscope cover glass (Thermo scientific, Gerhard Menzel GmbH, Braunschweig, Germany, 18 × 18 mm, 165027) and allow slides to dry in the dark.

### Confocal scanning microscopy and *z*-stack image acquisition

To reconstruct and quantify liver tissue, a confocal laser scanning microscope (Olympus, Germany, FV1000) was used and *z*-stacks were generated as follows:

1. Cover the tissue slice with one drop of Olympus immersion oil type-F (Olympus, Japan, IMMOIL-F30CC).
2. Prepare the *z*-stacks by 20× or 60× oil objectives (the settings are described in supplemental table 2).
3. Adjust the parameters of the scanning mode as described in supplemental table 2.
4. Select the corresponding dyes from the dye list. For architectural staining, select DAPI (blue; nuclear staining), alexafluor 488 (green; DPPIV/CD26 signal), Cy3 (white; GS staining) and Alexafluor 647 (red; DMs channel).
5. Stop the pre-scan mode and activate the 3D scanning mode.
6. Define the scanable first and the last *z*-levels.
7. Press the ‘start’ button to start manual recording, adjust the laser energy settings (HV, gain and offset) to approximately every 1 μm of *z*-level and record each level.
8. Press the ‘end’ button and start the automatic recording mode.
9. Deconvolute the 3D image using AutoQuant X3 (Bitplane). The 3D image is then ready for the pre-processing steps as described in the ‘Image Processing with TiQuant’ section.

## Image analysis protocols

The TiQuant software is used for image processing of multi-cellular tissues. It uses the open-source image processing and visualization libraries Insight Segmentation and Registration Toolkit (ITK) and Visualization Toolkit (VTK), as well as Qt, OpenGL/GLUT and HDF5. Similar to its precursor software (Hoehme and Drasdo 2010), TiQuant will become part of an open-source software framework which also includes modelling and simulation capabilities. This software framework is able to receive input directly from the image processing and analysis tool provided in this paper.

### Equipment setup

1. A computer (64 bit, preferably multi-processor, minimum of 16 GB RAM, 50 MB free disc space) with a Unix/Linux or Microsoft Windows operating system.
2. Installation of TiQuant, which is available from our homepage (www.msysbio.com/tiquant).
3. Installation of image processing software capable of reading and visualizing 3D image stacks (e.g. ImageJ).
4. Digitized image stacks in TIFF format prepared according to the above-described staining protocols comprising DAPI, DPPIV/CD26, GS and DMs channels, imaged using the 60× objective at a voxel resolution of 0.207 μm × 0.207 μm × 0.54 μm and 1,024 pxl × 1,024 pxl in *x*–*y* dimension. This set-up shows a fraction of one hepatic lobule.
5. Imaging set-ups meant for the analysis of a complete lobule should be imaged with a 20× objective at a voxel resolution of 0.621 μm × 0.621 μm × 0.54 μm and 1,024 pxl × 1,024 pxl.

### Image processing with TiQuant

The current version of TiQuant implements a number of so-called image processing pipelines tailored for the segmentation and quantification of veins, hepatic and non-hepatic nuclei, sinusoidal and bile canaliculi networks, as well as hepatocytes and necrotic tissue in 3D, which are obtained by confocal microscopy of liver lobules. These processing pipelines essentially are sequences of image filters and algorithmic units that can be parameterized to compensate for image feature variability. The following sections introduce all processing pipelines and their parameters in a way that will enable users not familiar with image processing to work with the software.

### Quick guide to TiQuant’s graphical user interface

The user interface of TiQuant is split into two functional panels.

In the left panel, the processing pipeline selection menu is located (first button in the left panel), as well as buttons for starting a pipeline, adding pipeline ‘jobs’ to a queue, and starting the latter.

In the right panel, a table is shown that allows for parameterization of the currently selected pipeline. Parameter values are editable by double clicking in the corresponding field in the ‘Value’ column. In case the parameter is a filename, a double click will enable a small button in the value field, which will open a file browser.

After adjustment of all parameters, a pipeline can be either started by clicking ‘Start pipeline’ or added to a processing queue by clicking ‘Add Job’ in the left panel. The latter will not start the pipeline immediately, but queue it for later sequential processing. When all jobs have been added, processing of the queue can be started by clicking the ‘Start queued jobs’ button.

### Workflow

To ensure segmentation and quantification of results that are of high and reproducible quality, it is necessary to follow an iterative procedure.

First, all channels of a dataset that are meant to be used for segmentation have to be thoroughly examined and assessed for quality. It is imperative that structures to be segmented are clearly visible and are set apart from the background throughout the whole dataset. If a lack of staining or high background noise compromises a considerably large part of the dataset, then the dataset may not be suitable for quantification. If the dataset is of sufficient quality for quantification, it is recommended that after pre-processing, to use the default parameter set of a processing pipeline be used to yield an initial segmentation. After execution, the quality of the result has to be assessed. For this purpose, each pipeline produces a file ending with the suffix ‘_overlay’, which is an overlay of the segmented structures over the raw data. In addition, the segmentation is saved as a binary mask with the file ending ‘_bin’, which may then be used as input for later pipelines. It is recommended that the overlay image is used for quality assessment. Besides the obvious quality criteria such as alignment of segmentations to underlying structures, pipeline-specific quality criteria are noted in the corresponding pipeline section. If a segmentation result is found deficient, the problematic filter in the pipeline has to be identified. For this purpose, each filter within a pipeline produces an intermediate result, which is named according to the filter. Based on these intermediate results, filter parameters have to be adjusted to improve segmentation quality. Afterwards, the pipeline has to be executed again, and the quality assessment and parameter adjustment procedure is iterated. It is advisable to move the results obtained from earlier attempts into a subdirectory in order to examine the effect of the parameter adjustments. Otherwise, the results of earlier attempts will be overwritten.

The described procedure may have to be repeated several times depending on the image quality and experience of the user.

### Procedure

#### Preparatory steps

1. Open the image stack with image processing software (e.g. ImageJ).
2. Split the channels.
3. Convert each channel to grey-value 8-bit pixel precision and save as a tiff file.

### Pre-processing

Contrast limited adaptive histogram equalization (CLAHE):

In most cases, image stack quality in terms of brightness and contrast can be improved by the application of an algorithm for contrast and brightness equalization and enhancement. The following steps should be applied to each of the channels that will be used for further segmentation steps (DAPI, DPPIV, DMs):

1. Select the pipeline ‘CLAHE’.
2. Specify the file location of the channel that has to be processed.
3. Use the default parameterization in an image stack set-up as described above. Include the following the parameters:
  3.1 ‘Histogram Window Size’ specifies the size of the window in 2D, respectively, the cuboid in 3D, in pixel/voxel for which an intensity histogram is compiled. The size should be larger than half the size of the features that are to be amplified. The larger the window, the longer the runtime.
  3.2 ‘Step Size’ specifies the rate in pixel/voxel at which a new histogram window is calculated along each axis.
  3.3 ‘Clip Level’ regulates the strength with which features are amplified. The parameter range is 0–1, where 1 means that the algorithm behaves similarly to the adaptive histogram equalization (AHE) and thus produces the strongest amplification. Lower values will limit unwanted noise amplification. Values of 0.1 or smaller are reasonable for many microscopy image set-ups, depending on the amount of background noise and intensity of features.
4. After execution, a file is created in the same folder with the same name as the input file with the suffix ‘_clahe’.

#### Crop

In most cases, several first and last *z*-slides contain imaging artefacts (e.g. out-of-focus blur) and have to be discarded. To do so:

1. Open all channels after CLAHE application with image processing software and identify the first and last *z*-slide that has no distortions in any channel.
2. Select the pipeline ‘Crop’.
3. Specify the file location of the pre-processed channel. This pipeline processes one channel at a time.
4. Enter the number of the first and last *z*-slide of sufficient quality in the fields ‘*z* start’ and ‘*z* end’. If image stacks differ in maximal *x* and *y* dimension from the default value, fill in the used dimension in ‘*x* end’ and ‘*y* end’.
5. After execution, a file will be created in the same folder with the same name as the input file with the suffix ‘_cut’.

##### Note:

Crop each channel of an image stack using the same x, y and z bounds.

##### Troubleshooting:

Normally, it is not necessary to apply CLAHE to the GS channel. However, if the GS channel has to be later used for vein segmentation, then it must be cropped.

### Segmentation

#### Necrotic region segmentation

1. Select ‘Segment Necrotic Region’ in the pipeline selection menu.
2. Specify file locations of pre-processed DPPIV and DMs channels. The DPPIV channel will be used for result visualization only.
3. Choose the threshold mode. The sample region size of the ‘Adaptive Otsu Threshold’ should be at least half the diameter of the largest necrotic region along the respective axis. The number of samples regulates at how many points within the dataset a sample region is constructed, and an individual Otsu Threshold is calculated. More samples increase the processing time. The larger the sampling region, the fewer sampling points are needed. An alternative to the ‘Adaptive Otsu Threshold’ is the ‘Manual Threshold’ option, where a threshold for the whole image stack can be manually specified.
4. The first erosion dilation pair is for noise removal and closing of small cavities within the necrotic region. The dilation kernel size should be roughly twice the size of the erosion kernel size. The kernel sizes depend largely on the level of noise within the dataset.
5. The second erosion dilation pair is for the removal of sinusoidal structures. Therefore, kernel size of the erosion operator should be slightly larger than the sinusoidal radius (in pixel). The kernel size of the dilation operator should be slightly larger than the erosion kernel to close the remaining cavities within the necrotic region.
6. Remove small isolated objects. In the preceding step, most of the sinusoidal system was removed. However, the complete structure may not have been removed by the erosion operator if very thick sinusoids and branching structures were present. Therefore, in this step, all objects below a certain volume will be removed. Adjust the volume, in case of very small necrotic regions (e.g. at dataset borders).
7. After execution using the DPPIV and DMs channel and files beginning with the prefix ‘necroticRegion’, the segmentation is saved as a binary mask and two overlay images.

##### Note:

 The boundaries of the segmented necrotic region should match the image data, as they will be used for distance measures. Holes within the segmented necrotic region are tolerable and sometimes inevitable due to the lack of DMs signal.

#### Vein segmentation

1. Select ‘Segment Veins’ in the pipeline selection menu.
2. Specify file locations of the pre-processed DPPIV, DMs and (optionally) DAPI channels.
3. Find for each channel an intensity threshold that delimits the lumen of the veins as completely as possible using image processing software.
4. The opening radius is determined by the radius of the lumen of the largest attached blood vessel that should not be part of the vein segmentation.
5. The closing radius is determined by the radius of the largest object in the vein lumen. The signals of macrophages, erythrocytes or other cells remaining in the lumen of the vein during imaging will thereby not compromise segmentation.
6. Select ‘Seed Point Selection’ below the parameter table to open the point selection window.
  6.1 Place a marker in the lumen of each central vein. Click ‘Finish CV Seed Point Selection’.
  6.2 Place a marker in the lumen of each portal vein. Click ‘Finish PV Seed Point Selection’.
  6.3 Click ‘Finish Seed Point Selection’. The seed point selection window will close, and the pipeline is ready to be started.
7. After execution, the segmentation of central and portal veins is saved as a binary mask and overlay images in files beginning with the prefix ‘vein_central’ and ‘vein_portal’, respectively.

##### Critical step:

Seed point selection window handling:

1. Browse the stack using the up and down arrow keys. In the lower left corner, the slide number is shown.
2. Enter/Quit a seed point marker input mode by pressing the ‘s’ key.
3. While in input mode, seed points can be positioned at the image plane using the left mouse button. Placed seed points can be dragged. The last placed seed point can be deleted by pressing the ‘Delete’ key.

##### Troubleshooting:

It is crucial to identify the appropriate thresholds. Use thresholds that define the margin of the lumen as completely as possible. Two, if necessary three, channels can be used to reconstruct the veins. Holes in the endothelium that are present in all two/three threshold channels may result in erroneous reconstruction of veins, because boundary information is missing. In this case, larger opening and/or smaller closing radii may help.

##### Note

Boundaries of the segmented veins must match with the image data, as they will be used for distance measures.

### Sinusoid and Bile Canaliculi segmentation

1. Select ‘Segment Sinusoids + Bile Canaliculi in 60× Datasets’ in the pipeline selection menu.
2. Specify file locations of the pre-processed DPPIV and DMs channels. In case the image stack contains necrotic tissue, check the ‘Is there a necrotic region’ box and specify the necrotic region segmentation file. Adjust voxel spacing settings if the image set-up differs from the default values.
3. Select threshold modes for segmentation of sinusoids in DPPIV and DMs channels. Recommended is the ‘Adaptive Otsu Threshold’. Some datasets may require the specification of a manual threshold. A voxel needs to pass thresholds in both channels to be considered a sinusoidal voxel, which helps minimize false positives. However, locally impaired intensity of one channel may prevent correct segmentation; therefore, careful threshold tuning is mandatory.
4. Remove noise artefacts. For the removal of very small artificial objects due to ‘salt-and-pepper noise’ an inverse hole-filling operator is applied. Radii should not exceed 3 pixel. Majority threshold values have to be within the range of 0–10. The smaller the value, the stronger the noise reduction effect, where values smaller than 2 may also remove some boundary voxels of larger objects.
5. Fill cavities within sinusoids. It is recommended to use the accelerated (and less precise) version of the algorithm. Set the radius to a value slightly larger than the largest cavity. In case there are holes that are not completely surrounded by segmented voxels, increase the ‘minimal fraction of surrounding foreground’ value and decrease this value once artificial contacts are introduced. It is recommended to use the ‘accelerated’ version.
6. Close the remaining cavities and discontinuities and remove small artificial objects. The closing radii should equal the largest remaining cavity radii. Opening radii should be slightly larger than radii of the largest artificial objects.
7. Remove isolated objects. All isolated objects of a volume smaller will be removed.
8. Remove noise in the DPPIV channel prior to the bile canaliculi segmentation. In case of high noise levels in the DPPIV channel, the median and greyscale opening radii might be increased to a maximum value of 2.
9. Select a threshold mode for segmentation of bile canaliculi in DPPIV channel. Recommended is the ‘Adaptive Otsu Threshold’. Some datasets may require a manual threshold. Since in this step the focus is on the bile canaliculi, the method and/or value may differ from the one used in step 4.
10. Close cavities and small discontinuities. The radii of the hole-filling operator should not exceed 2. The majority value should be smaller than 4 to ensure the closing of very small gaps. Smaller majority values may result in additional boundary voxels. Make sure that branching points are still clearly defined.
11. Removal of artificial objects. There are three steps in the noise removal process. The opening may affect segmented bile canaliculi if their radius is in the range of the opening radius. In this case, skip the opening by using the default radius value of 0. Small artificial objects will be removed by inverse hole filling. Proceed as in step 5. The final step in object removal will discard all objects with a volume smaller than the specified value.
12. After execution, the segmentation of sinusoidal and bile canaliculi networks is saved as binary mask and overlay images in files beginning with the prefix ‘sinus’ and ‘bile’, respectively.

#### Note:

It is critical that the segmented sinusoids and bile canaliculi appear as solid structures free from inner holes. Otherwise, the network parameters will be altered. Segmentation boundaries have to match the endothelial staining in the DPPIV/DMs channel as precisely as possible. Pay special attention to the connectivity of the bile canaliculi network. Limit the number of artificial branch gaps, especially at intersections, to a minimum. Otherwise, the number of dead-end branches and intersections may be increased or decreased.

### Network graph extraction and analysis

1. Select ‘Extract and Analyze Graph’ in the pipeline selection menu.
2. Choose the network type from the drop-down menu. This is a convenience option that will determine the network type-dependent default parameters.
3. Choose the type of input from the drop-down menu. The ‘Skeleton Image’ option will transform a skeleton image of a network into a graph representation and optionally visualize and analyse it. The ‘Graph’ option skips the transformation steps.
4. Depending on the input type, specify the file location of the skeleton image or the first graph file (ending on the suffix ‘_graph0.txt’).
5. Validate this for both network types using the following parameters:
  5.1 Resampling filter: The transformation of a network skeleton into a network graph produces a graph where vertices represent single voxels. Thus, graph edges have a length of single voxels. The re-sampling factor determines the fraction of vertices per branch that are kept. Intermediate vertices will be discarded. This procedure prevents the quantification of artefacts.
  5.2 Remove dead-end filter: All dead-end branches of a length smaller than the threshold are considered artificial and will thus be discarded.
  5.3 Collapse intersection nodes filter: All intersection node paired within a distance smaller than the threshold are considered artificially separated and will thus be merged together.
  5.4 Geometric pruning filter: Vertices that introduce a deviation from a straight line of less than this angle are considered obsolete for maintaining the geometrical structure of the graph and will thus be deleted. Instead of this vertex and the two attached edges, a direct edge between the two other edge defining vertices is introduced. This procedure is applied to vertices with exactly two attached edges.
6. Select whether an analysis of the network graph is desired.
7. The ‘Output file prefix’ will be used as a prefix for the analysis files. It is recommended to use the default value.
8. Specify the dataset name. All quantified objects will contain a reference to the dataset name in the analysis file.
9. Specify the file locations of the requested segmentation files.
10. Specify the voxel size in case it differs from the default values.
11. Pipeline execution graphs will be visualized, if the ‘Display Options’ are set accordingly. Graphs are saved in text files beginning with the prefix from point 7. Sub-graphs that are not connected are stored in individual, enumerated files.

### Nuclei segmentation

1. Select ‘Segment Nuclei in 60× Datasets’ in the pipeline selection menu.
2. Specify file location of the pre-processed DAPI channel. Adjust the voxel spacing according to the image setup.
3. In case image data have high noise levels, increase the median filter and greyscale opening radii.
4. Choose a threshold mode. ‘Adaptive Otsu Threshold’ will yield superior results in most cases. In case of high noise levels or weakly stained structures of interest, the results of the Otsu method may not be satisfying quality. Try to find a ‘Manual Threshold’ instead.
5. Remove ‘salt-and-pepper noise’ using ‘Inverse Hole Filling’. For high ‘salt-and-pepper noise’ levels, lower the ‘Majority Threshold’ and increase the radii. For low noise levels, the ‘Majority Threshold’ may be increased.
6. Close cavities in nuclei. Set the radius to a value slightly larger than the largest cavities. In case of holes that are not completely surrounded by segmented voxels, increase the ‘minimal fraction of surrounding foreground’ value and decrease this value if segmentation suffers from erroneously enlarged nuclei. It is recommended to use the accelerated version.
7. Close any last remaining cavities that are not completely surrounded by segmented voxels. Closing radii should be at least equal to the largest cavity radii. Too large radii may result in the artificial merging of separated objects.
8. Remove small artificial objects that are few voxels in size using the opening operator. Too large opening radii may result in the artificial separation of objects.
9. Separate the artificially agglomerated nuclei. The ‘alpha’ default value will be appropriate in most cases. Smaller alpha values will result in more, whereas larger alpha values will result in less separation events.
10. Remove artificial objects and group nuclei. In this step, each object will be evaluated according to its diameter. All objects with a diameter smaller than ‘smallest non-hepatocyte diameter’ will be dropped. Remaining objects are classified according to their diameter as non-hepatic or hepatic nuclei. In case the ‘biggest non-hepatocyte diameter’ exceeds the ‘smallest hepatocyte diameter’, nuclei with a diameter in this overlapping range will be classified according to their roundness measure, which ranges from 1 for spherical objects to 0 for infinitely elongated objects.
11. After execution, the segmentation of hepatic and non-hepatic nuclei is saved as binary mask and overlay images in files beginning with the prefix ‘hepNuclei’ and ‘non-HepNuclei’, respectively.

#### Note:

 Segmented nuclei should contain no inner holes and must be separated and classified correctly. Separation of nearly spherical agglomerations of nuclei may fail in some cases.

### Hepatocyte shape approximation

1. Select ‘Approximate Cell Shape’ in the pipeline selection menu.
2. Specify the dataset file list, ending with ‘.ias’. This will automatically fill in most of the necessary segmentation files. Fill in the DPPIV channel, check the necrotic region check box if necessary and fill in the voxel spacing.
3. It is advisable to use the default parameterization in an image stack set-up as described above. Adjust the following:
  3.1 ‘Bile weight’ is a measure which balances the influence of bile canaliculi and sinusoids on the one hand and nuclei on the other, with respect to the cell shape approximation. The parameter range is 0–1, with 0 representing full emphasis on nuclei, ignoring the bile canaliculi and sinusoids, and 1 representing the contrasting situation.
  3.2 As in the nuclei segmentation pipeline, the ‘alpha’ regulates the number of objects that will be produced. Smaller alpha values will result in more, whereas larger alpha values will result in less individual cells.
  3.3 Cells without nuclei and cells with diameters smaller than ‘Minimal cell diameter’ are considered artificial and will be discarded.
4. After execution, the cell shape approximation results are saved as binary mask and overlay images in files beginning with the prefix ‘cellShape’.

#### Note:

 It is critical that boundaries of approximated hepatocytes align with confining structures. Artefacts in sinusoid or bile canaliculi segmentation may lead to incorrect cell boundaries. At dataset borders, cell boundaries may be incorrect due to incomplete confinement information. These artefacts can be ignored, since only inner cells that are not in contact with dataset borders are used for quantification.

### Hepatocyte analysis

1. Select ‘Analyze Cells’ in the pipeline selection menu.
2. Specify the dataset name. All quantified objects will hold a reference to the dataset name in the analysis file.
3. Specify the dataset file list ending on ‘.ias’. This will create a parameter table with all necessary segmentation files.
4. In case of a different voxel size, amend the voxel spacing parameters.

### How to handle data with different image settings

The effective voxel size of our datasets was 0.207 μm × 0.207 μm × 0.54 μm. The default parameter values of all pipelines are dependent on this dataset set-up.

In case the voxel size differs due to e.g. different magnification or voxel resolution, all kernel values have to be rescaled. For example, a closing operator using a kernel of size 2, 2, 1 in our set-up would use a rescaled kernel of size 4, 4, 1 in a set-up with voxel sizes of 0.414 μm × 0.414 μm × 0.54 μm. However, this rule of thumb is only applicable when the feature appearances of all structures of interest to be segmented are similar in appearance to our setup. If this prerequisite is not met (e.g. due to lower magnification where sinusoids appear as solidly stained structures, rather than stained endothelial cells that encircle a stain-free lumen), necessary parameter adjustments might be less straightforward; and in extreme cases, the pipelines may not be applicable.

### Modelling based on TiQuant

In the upcoming open-source software framework CellSys, the quantifications obtained from TiQuant can directly be used as an input for the CellSys modelling engine. As an illustration of this application of the processing pipeline, we refer to a model of liver regeneration after CCl_4_-induced liver damage. This model correctly predicted a previously unknown and subsequently experimentally validated order mechanism that was found essential for liver regeneration (Hoehme et al. PNAS 2010).

### Tissues of mouse, human and pig liver

Mouse liver tissues used for the current stainings were obtained from: (1) C57Bl6/N mice, 8–12 weeks old, male (Charles Rivers, Sulzfeld, Germany) and (2) DPPIV/CD26-deficient mice were generated by re-derivation of mice (C57BL/6-DPPIVtm1Nwa/Orl) from a frozen stock at the European Mouse Mutant Archive (EMMA). The gene was inactivated by homologous recombination resulting in homozygous mice on the C57BL/6 background with inactive CD26 genes, both soluble and membrane bound (Marguet et al. 2000); (3) β-catenin KO mice as described in our previous publication (Braeuning et al. 2010); and (4) the livers from 12-week-old BALB/c-Abcb4−/− (MDR2 KO) animals were used to analyse the hepatic stellate cell markers. Pig liver tissue was obtained from 10- to 14-week-old female piglets (Large White, common land race pigs, from INRA Jouy-en-Josas, France) weighting 28–42 kg at the time of 70 % liver resections. Human liver was obtained from the University Hospital of Jena. The local authorities have approved the use of liver tissue from mice and pigs. The local ethical committee has approved analysis of human liver tissue for immunostaining.

## Electronic supplementary material

Below is the link to the electronic supplementary material.

Supplementary material 1 (DOCX 5164 kb)
