# Supplementary material for: Protocols for staining of bile canalicular and sinusoidal networks of human, mouse and pig livers, three-dimensional reconstruction and quantification of tissue microarchitecture by image processing and analysis
Source: Arch Toxicol. 2014 Apr 19;88(5):1161–83. doi: 10.1007/s00204-014-1243-5 (PMC3996365; doi:10.1007/s00204-014-1243-5)

**Supplemental fig. 1:** **Further primary antibodies that can be used with the standard ‘architectural staining’ protocol.** The standard protocol can be used with the exception that the first antibodies have to be exchanged using the conditions summarized in supplemental tables 1A and 1B. α-SMA: alpha-smooth muscle actin; α-tubulin: alpha-tubulin; ICAM1: intercellular adhesion molecule-1; lectin: biotinylated *Solanum tuberosum* lectin; LDL-R: low-density lipoprotein-receptor; Mrp2: Multidrug resistance-associated protein 2.

**Supplemental fig. 2:** **Imaging of stellate cells. A)** and **B)** staining of desmin visualizes stellate cells in the active and quiescent state in healthy liver tissue **(A)** desmin positive stellate cells are located along the sinusoids. After induction of liver damage by CCl_4_ a strong accumulation of desmin positive cells **(B)** in found in necrotic areas. a murine liver upon CCl_4_ intoxication. Scale bars are 20 μm. **C)** and **D)** alpha-smooth muscle actin (α-SMA) serves to visualize activated stellate cells. In healthy liver tissue **(C)**, no α-SMA positive stellate cells can be observed. Only the endothelial cells of vessels appear positive. After induction of liver damage by CCl_4_ α-SMA positive stellate cells **(D)** are observed in the damaged pericentral areas. Scale bars are 100 μm. **E)** In fibrotic livers, e.g. after MDR2 knockout, α-SMA positive stellate cells are observed in the fibrotic streets. Scale bar is 100 μm.


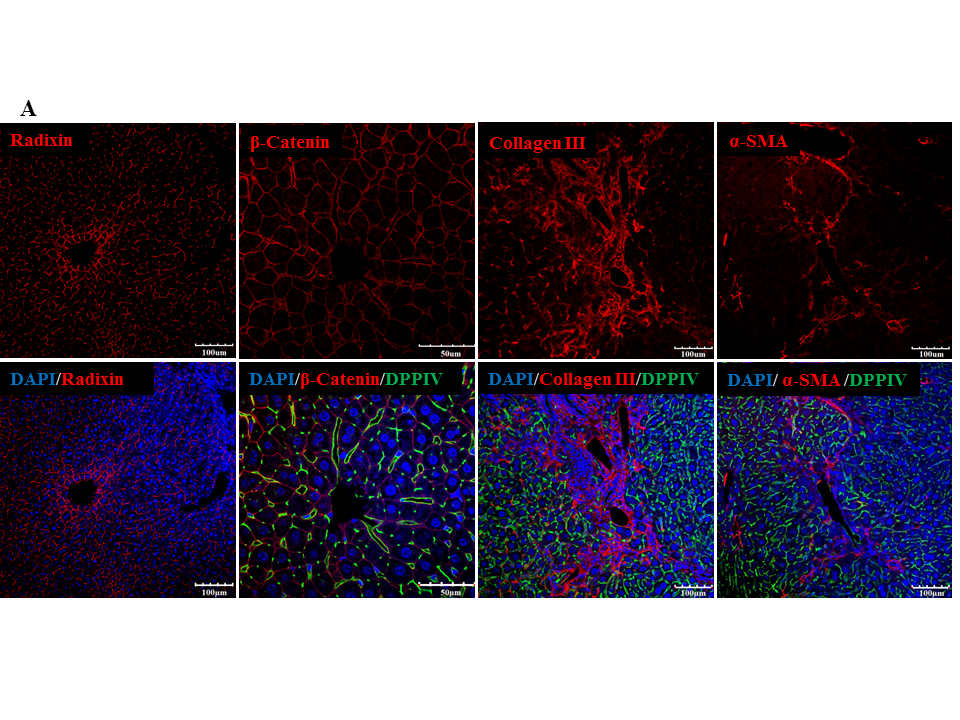


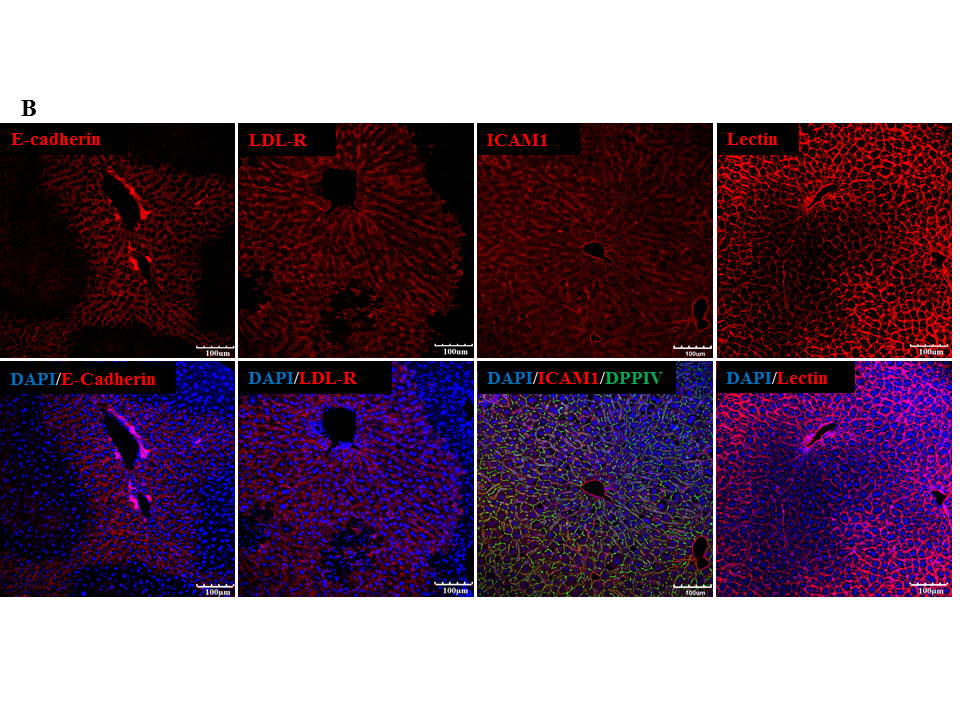


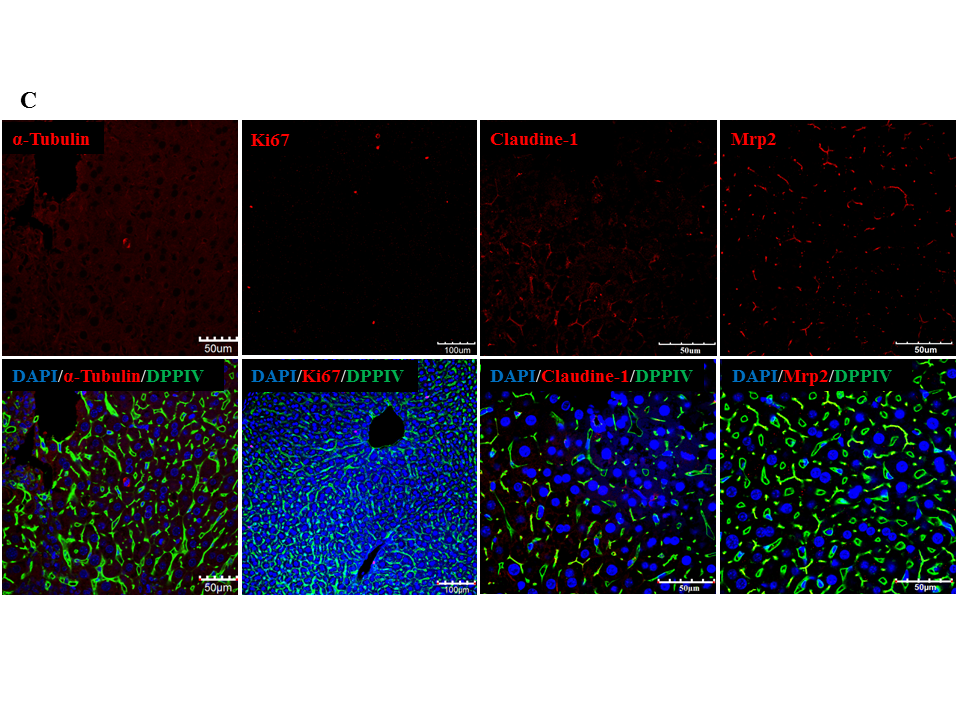


**Supplemental figure 1**

**
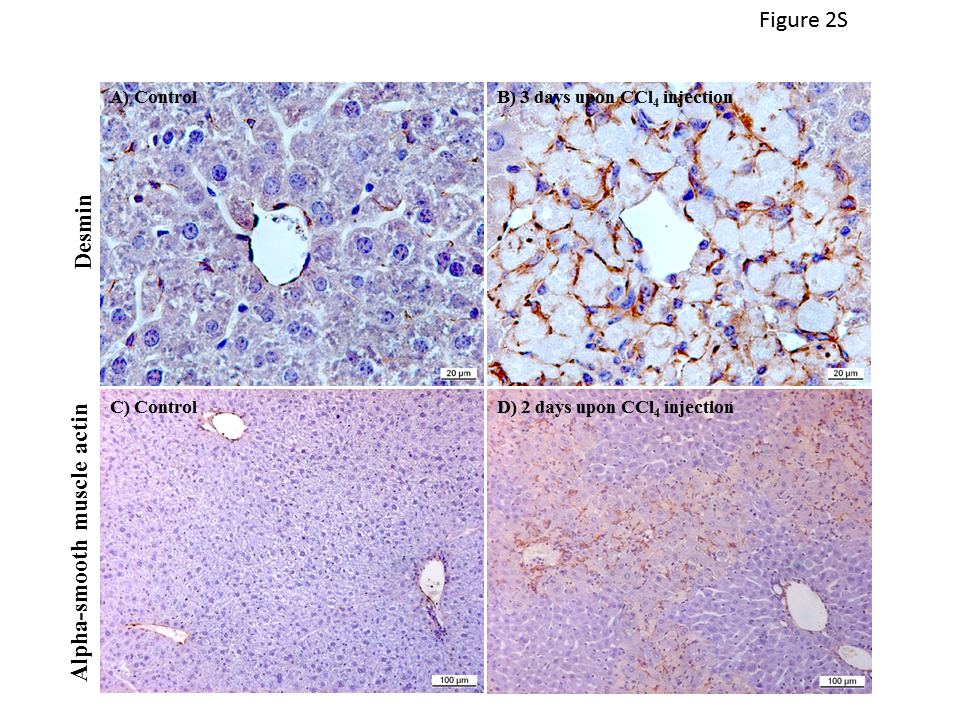
**

**
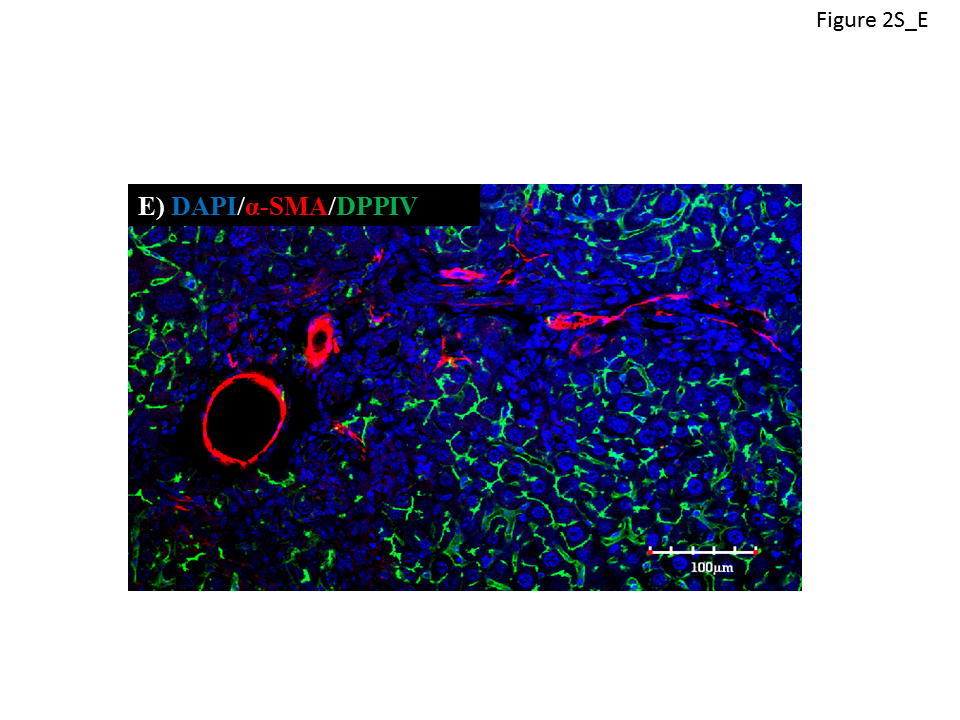
**

**Supplemental figure 2**

**Supplemental Table 1:** **List of antibodies that can be sued in the ‘architectural staining’ protocol.** Primary **(A)** and secondary **(B)** antibodies together with information on ordering, dilution and target species are included.


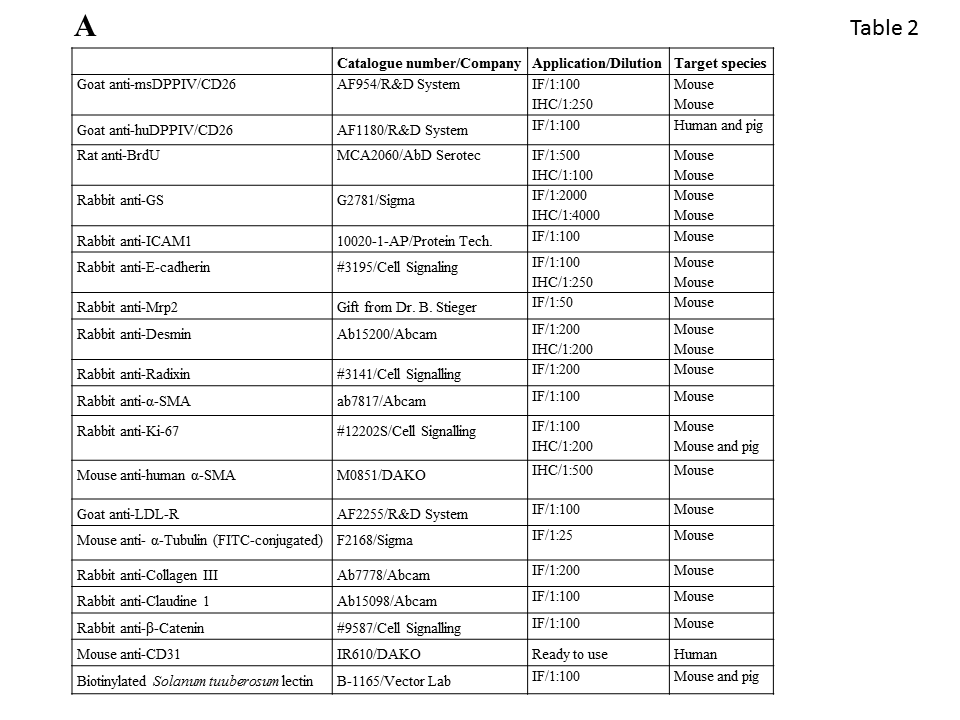


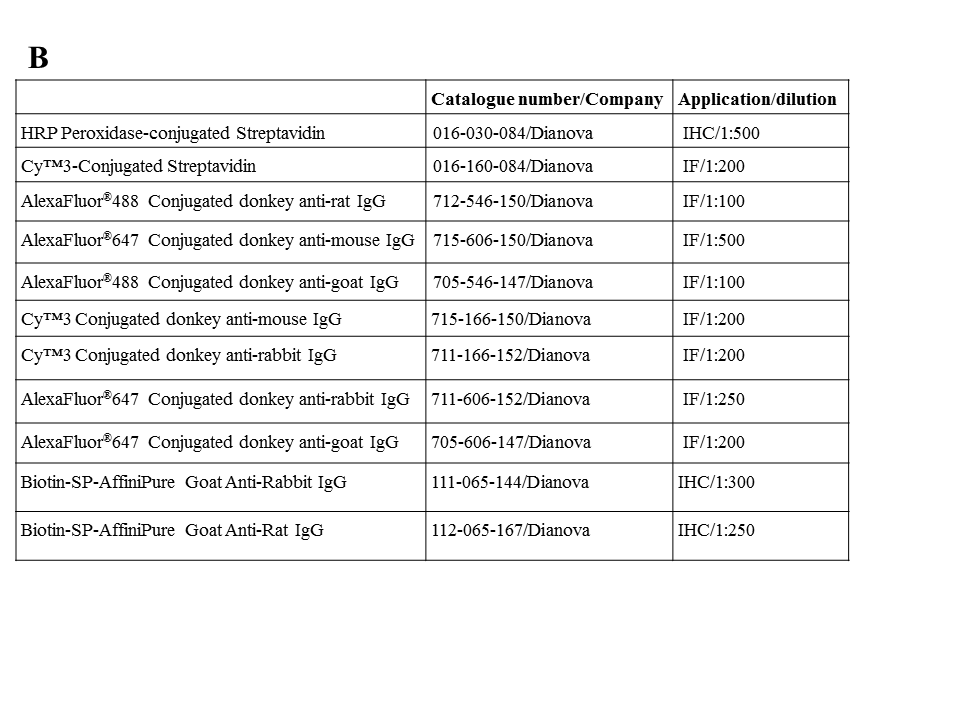


α-SMA: alpha-smooth muscle actin; α-tubulin: alpha-tubulin; BrdU: bromodeoxyuridine; DPPIV: dipeptidyl peptidase IV; ICAM1: intercellular adhesion molecule-1; IF: immunofluorescence; IHC: immunohistochemistry; GS: glutamine synthetase; LDL-R: low-density lipoprotein; Mrp2: Multidrug resistance-associated protein 2.

**Supplemental Table 2:** **The settings of both 20x and 60x objectives.** It should be considered that the pinhole settings are automatically adjusted.


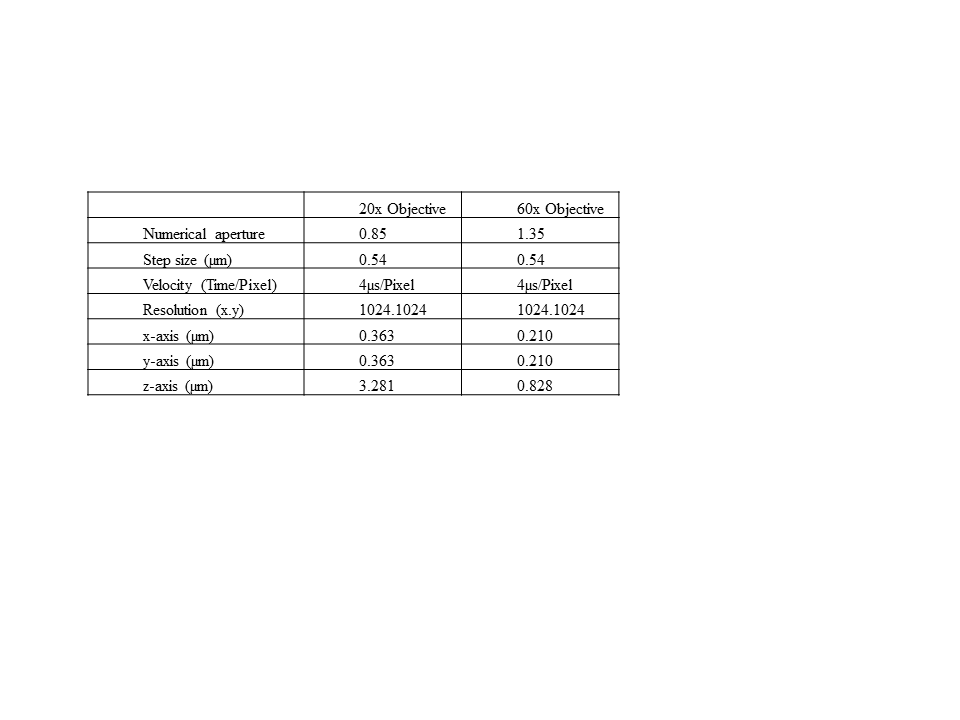

Supplement: Supplementary file 1 — Supplementary material 1 (DOCX 5164 kb) [file 204_2014_1243_MOESM1_ESM.docx]
